# Language training for oral and written naming impairment in primary progressive aphasia: a review

**DOI:** 10.1186/s40035-021-00248-z

**Published:** 2021-07-16

**Authors:** Ilaria Pagnoni, Elena Gobbi, Enrico Premi, Barbara Borroni, Giuliano Binetti, Maria Cotelli, Rosa Manenti

**Affiliations:** 1 Neuropsychology Unit, IRCCS Istituto Centro San Giovanni di Dio Fatebenefratelli, Brescia, Italy; 2 Vascular Neurology Unit, Department of Neurological and Vision Sciences, ASST Spedali Civili, Brescia, Italy; 3 Neurology Unit, Department of Clinical and Experimental Sciences, University of Brescia, Brescia, Italy; 4 MAC Memory Clinic and Molecular Markers Laboratory, IRCCS Istituto Centro San Giovanni di Dio Fatebenefratelli, Brescia, Italy

**Keywords:** Agrammatic variant of primary progressive aphasia, Semantic variant of primary progressive aphasia, Logopenic/phonological variant of PPA, Naming

## Abstract

**Background:**

Primary progressive aphasia (PPA) is a neurodegenerative disorder characterized by a gradual, insidious and progressive loss of language abilities, with naming difficulties being an early and persistent impairment common to all three variants. In the absence of effective pharmacological treatments and given the progressive nature of the disorder, in the past few decades, many studies have investigated the effectiveness of language training to minimize the functional impact of word-finding difficulties in daily life.

**Main body:**

We review language treatments most commonly used in clinical practice among patients with different variants of PPA, with a focus on the enhancement of spoken and written naming abilities. Generalization of gains to the ability to name untrained stimuli or to other language abilities and the maintenance of these results over time are also discussed. Forty-eight studies were included in this literature review, identifying four main types of language treatment: a) lexical retrieval treatment, b) phonological and/or orthographic treatment, c) semantic treatment, and d) a multimodality approach treatment. Overall, language training is able to induce immediate improvements of naming abilities in all variants of PPA. Moreover, despite the large variability among results, generalization and long-term effects can be recorded after the training. The reviewed studies also suggest that one factor that determines the choice of a particular approach is the compromised components of the lexical/semantic processing system.

**Conclusion:**

The majority of studies have demonstrated improvements of naming abilities following language treatments. Given the progressive nature of PPA, it is essential to apply language treatment in the early stages of the disease.

## Introduction

Originally described as a nosological entity, primary progressive aphasia (PPA) is now considered a neurological syndrome characterized by a variety of clinical symptoms with different degrees of severity, including speech and language impairment due to the degeneration of language networks [1, 2]. The loss of language abilities is gradual and insidious, and speech becomes progressively slower and deconstructed in its phonological, semantic or syntactic aspect [1, 3–5].

For a diagnosis of PPA, language impairment should be the most prominent deficit, at least for the first 2 years of symptom onset, without other cognitive and behavioural disorders [1, 6, 7]. Moreover, the main cause of difficulties in daily life activities is the progressive deterioration of language cortical areas and/or networks involved in word production or comprehension [1, 6].

Interest in PPAs has grown rapidly over the years [2]. Recent guidelines for the classification and diagnosis of PPAs have identified the following variants of PPA: nonfluent/agrammatic variant (nf/avPPA), semantic variant (svPPA), and logopenic/phonological variant (l/phvPPA) [6].

The nf/avPPA has main clinical features of agrammatism in language production and/or effortful halting speech [6]. As an initial sign of disease, apraxia of speech is frequently recorded [3, 6, 8]. Due to difficulties in motor speech planning, patients typically display speech sound errors and altered prosody. Variable degrees of anomia, as well as an impairment of syntactically complex sentence comprehension, are also evident [2, 3, 6, 7]. From the neuroimaging perspective, this disability is characterized by the left inferior fronto-insular atrophy and the involvement of white-matter fiber bundle in the dorsal language pathway [3, 9–11].

The svPPA is clinically characterized by a slow loss of semantic knowledge [7], anomia, and single-word comprehension deficits, especially for low-frequency items [6, 7, 12]. The impairment in single-word comprehension is usually the earliest symptom of semantic memory deficit, subsequently leading to deterioration in recognizing objects and people [13]. Patients with svPPA are fluent in spontaneous speech and word repetition is spared, although they can exhibit surface dyslexia and dysgraphia [3, 6, 12]. The neuroanatomical profiles of svPPA include bilateral, although mainly left-lateralized, anterior inferior and mesial temporal lobe atrophy [13].

The l/phvPPA has core features of deficits in word retrieval and sentence repetition [6]. l/phvPPA patients have a slow rate of spoken language, which is characterized by simple sentences with frequent pauses due to difficulties in word-finding, and they do not show frank agrammatism. Single-word comprehension is relatively spared [6, 14]. The left inferior parietal lobule and the left posterior temporal lobe are consistently involved in the neurodegenerative process in l/phvPPA [2, 6, 14].

Although a detailed description of the distinctive features of PPA variants has been outlined, some patients show an aphasic syndrome that cannot be classified into any of the three variants [15]. A minority of patients present mixed features or a single language symptom (such as anomia or dyslexia) for a long time [6, 16]. In this case, due to the progressive nature of the disorder, the patients could fulfil the root criteria for PPA but not the criteria for any of the variants [15, 16]. Therefore, in addition to the three most common variants, the consensus criteria suggest that these patients should be categorized into “PPA unclassifiable” [6, 15, 16].

In a substantial number of patients with PPA, the disease arises at middle age and has a devastating effect on their quality of life [17]. Moreover, language impairment in PPAs is the main stressor for patients and their caregivers, which causes serious impacts on patient relationships, social networks, and the possibility of participating in a large number of everyday activities based on communication [12, 18].

In the absence of effective pharmacological therapies to improve or stabilize the cognitive-linguistic deficits, there has been an increased interest in language training interventions to minimize the functional impact of communication difficulties in daily life and remediate language deficits in PPAs [13, 17, 19, 20]. Currently, different interventions have been described for individuals with PPA [12, 17, 21, 22].

With the progress in the diagnosis of PPA, an important aspect for obtaining satisfactory results is to apply an impairment-based approach that focuses on the particular language deficits of a patient [23]. Several studies have suggested that word retrieval treatments, such as semantic, phonological and orthographic interventions, provide substantial benefits for naming abilities of PPA patients and often show a generalized effect on functional communication [24–26]. Other studies have focused on more specific interventions aimed at improving fluency in patients with nf/avPPA. In this regard, the script-training approach has been reported to ameliorate fluency, speech production and intelligibility of oral output in nf/avPPA [23, 27]. Furthermore, some authors have proposed communication skill training aimed at learning strategies or facilitative behaviour directed to successful daily communication [28, 29].

It is important to note that the use of the same type of language training in patients with similar clinical features may often lead to different results [21, 25, 30]. This suggests that when selecting the interventions to be applied, not only an accurate and detailed assessment of language and cognitive deficits is needed, but variables that could affect the outcome, such as motivation, the presence of a caregiver, the severity of the disease and the presence of anosognosia, should also be taken into account. Likewise, it is important to set realistic goals for language treatment based on patients’ needs [23]. Educational and support groups directed to caregivers have been shown to be of crucial importance too [31].

## Naming deficits in the variants of PPA

Naming difficulty is one of the first symptoms of PPA [3, 6, 17, 32]. Early detection of naming difficulties facilitates early intervention, thereby reducing their functional impact on everyday life.

Word-finding difficulties recorded in the three variants of PPA involve different linguistic processes, leading to substantial differences in the type of errors made by patients with PPA [33–35]. The word-finding process occurs through a multistep organization, from access to semantics of words to the retrieval of phonological code for oral naming or of orthographic code for written naming to the articulation process. This language model postulates that every step involves an extensive network of cerebral regions, predominantly lateralized in the left hemisphere of the brain [32, 36].

In PPA, the neural degeneration process can affect these language-related cortical regions differently, implying clinical and anatomical heterogeneity among the three variants [3, 37, 38]. Thus, it can be assumed that distinct components of the lexical/semantic processing system are involved in different naming difficulties.

For nf/avPPA, in which imaging studies have highlighted the involvement of the left posterior frontal and insular regions, patients have been reported to have naming deficits specifically during the naming of actions rather than of objects [39, 40]. In this case, anomia reflects damage to the post-lexical level of word production (phonological or orthographic) instead of to the lexical/semantic processing level [39, 41–44]. Impairment at this stage of naming process seems to impact phoneme selection during phonological encoding, so patients with nf/avPPA often show phonemic errors (deletions, substitutions, insertions and transpositions) [45]. In addition, nf/avPPA patients also present phonetic errors caused by progressive motor speech impairment (dysarthria and/or apraxia of speech) [33, 45, 46]. Object naming difficulties are predominantly evident in svPPA, while action naming is relatively preserved. For the svPPA variant, impairment at the semantic level of the naming process is the core feature of word-finding difficulties [17, 47, 48]. The type of errors systematically shown by svPPA patients reflects the progressive loss of semantic representations. Initially, errors are more evident in low-frequency and/or low-familiarity items, therefore they tend to replace the target with more familiar words or with superordinate category names. However, with the progression of the disease, the naming difficulties extend to high-frequency or high-familiarity words. Another typical phenomenon in the svPPA variant is the semantic paraphasias and the use of circumlocutions, both in naming tests and in spontaneous speech [16, 49, 50]. Semantic impairment in svPPA has been shown to be related to the left-sided anterior temporal atrophy/hypometabolism [51], whereas naming deficits are associated with degeneration in the superior regions of the left temporal pole [6, 52]. Moreover, in the l/phvPPA variant, word retrieval impairment is widely described [3, 16]. In the l/phvPPA condition, the naming difficulties may be caused by a deficit in lexical-phonological processing, leading to phonemic paraphasias [53]. In the l/phvPPA variant, the naming difficulties may correlate with atrophy/hypometabolism in the left posterior superior, posterior middle and middle inferior temporal cortex [6, 52, 54].

All of this evidence converges on the assumption that the anatomical differences underlying naming impairments in PPA reflect the involvement of separate word-finding operations, which, in turn, leads to different error types in naming tasks.

## Naming treatment in PPA

Several studies have demonstrated effectiveness of intervention approaches in improving naming difficulties in PPAs [12, 17, 22, 24, 25, 55–60], supporting both crucial roles of early intervention and the possibility of inducing generalization and long-term maintenance of gains [21, 56, 61, 62].

Due to the impact of language impairments on daily life activities, several studies have investigated the effectiveness of different speech-language therapies in providing word relearning interventions, strategies and aids for adequate communication and support, and providing education for caregivers [17, 63, 64]. As already described, naming impairment in PPA may reflect breakdown at one or more stages of the naming process [33–35]. In line with these considerations, several studies have investigated the effectiveness of naming treatments, including semantic, lexical-phonological and lexical-orthographic interventions and/or the combination of them, in patients with progressive word-finding difficulties [21, 25, 60], but only a few studies have compared the effects of different approaches in the same PPA-subtype patients [65–74].

In the following sections, we will discuss the immediate benefits of language training on the measures of oral and written naming outcomes, the possible generalization to other tasks, and maintenance over time for each variant of PPA.

## Search strategies and study selection criteria

We searched the Medline (PubMed) database for literature containing the following terms: *“(primary progressive aphasia OR semantic dementia OR frontotemporal dementia) AND (language training OR language treatment OR anomia training OR treatment for lexical retrieval OR anomia treatment OR word retrieval therapy)”.*

**Fig. 1:**
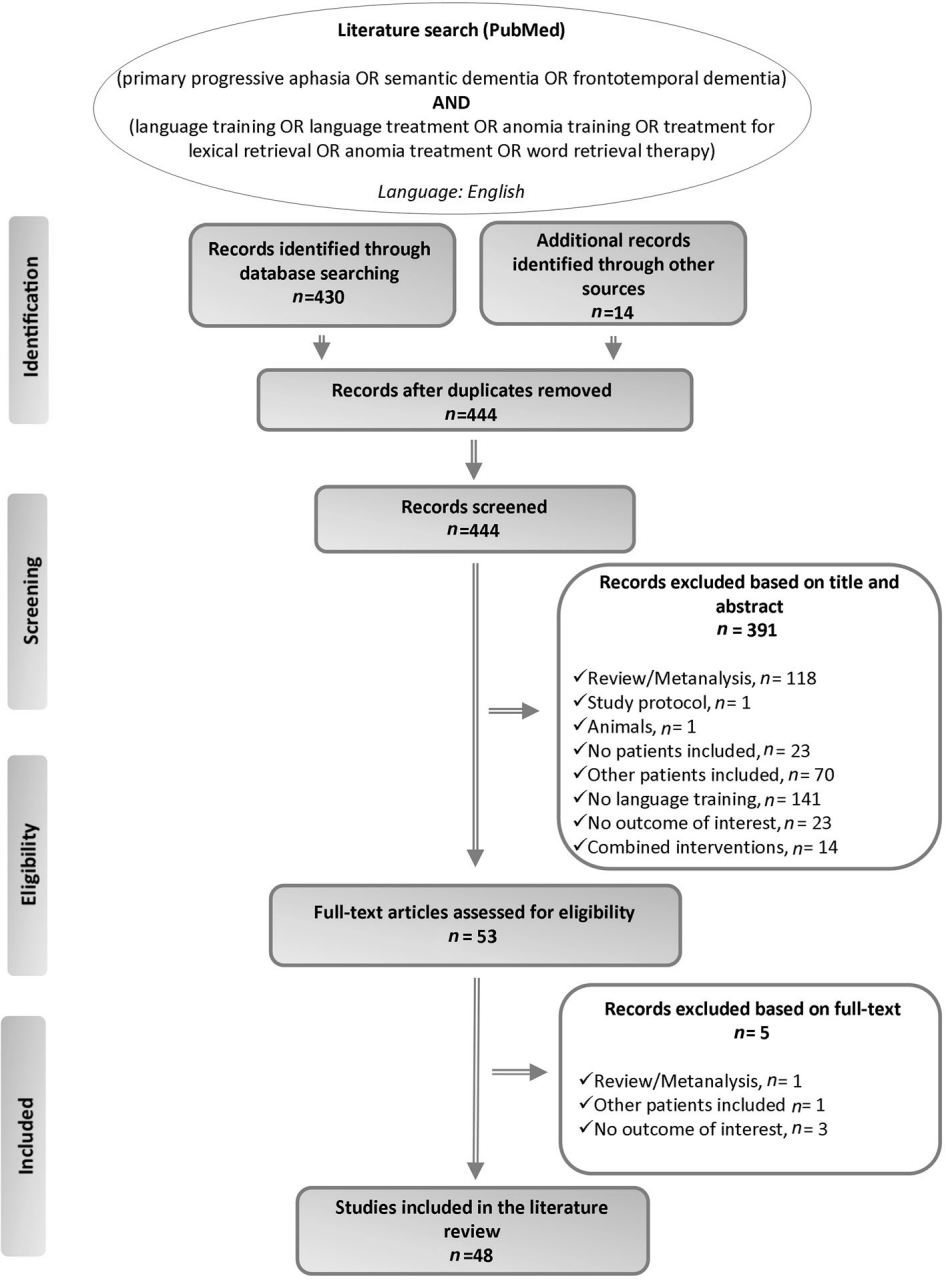
Summary of literature search – PRISMA flow diagram

We reviewed all titles and abstracts and examined all relevant original research articles (see flowchart in Fig. 1).

The results were screened by reviewing titles and abstracts and studies that met the following criteria were included: (a) original research; (b) conducted on patients with PPA; (c) comprising only behavioural language intervention and no combined interventions; (d) providing at least one oral or written naming outcome measure; and (e) published before 15 January 2021. Furthermore, animal studies, reports of secondary data such as meta-analyses, reviews or letters and study protocols were excluded. Full-texts of the remaining studies were read before their exclusion or inclusion. References of the included studies were also screened to identify possible additional sources. Only English-language articles were included. Finally, 48 studies published between 2001 and 2020 were included in this literature review (Fig. 1). The selected studies comprised 278 patients with a diagnosis of PPA (Table 1).

**Table 1:** Review of studies that assessed the effects of language training on oral and written naming abilities in patients with primary progressive aphasia

Study	Number of patients	Age, Mean (SD)	Protocol design	Language intervention and duration	Follow-up (weeks)	Outcome measures	Results
Lexical retrieval treatment (LRT)							
Frattali and Kang, 2004 [80]	1 svPPA 2 post-stroke aphasia	svPPA: 66 post-stroke aphasia: 65 (8.0)	Single-case pre-post design, treatment as within-subject factor	LRT using an errorless and effortful approach Duration: 9–12 sessions (120 min, 8–12 weeks)	24	Oral naming: - Naming of trained nouns and verbs (whole words) - Naming of untrained nouns and verbs (whole words)	Oral naming results in svPPA: - Improvement in naming for both trained nouns and verbs after errorless and effortful approach - No improvement in naming for both untrained nouns and verbs Gains not maintained at follow-up.
Jokel et al., 2009 [79]	2 nf/avPPA	66.5 (8.5)	Case series pre-post design	LRT Duration: 12 sessions (60 min, 3 weeks)	4 and 24	Oral naming: - Naming of trained nouns (whole words) - Naming of untrained nouns (whole words) - Naming of untrained nouns from PNT (whole words) Generalization measures: - syntactic generation task	Oral naming results: - Improvement in naming for trained items - No improvement in naming for untrained items - No improvement in naming for untrained items from PNT Generalization: - Syntactic generation task Gains maintained at 4-week follow-up: - Improvement in naming for trained items Gains not maintained at 24-week follow-up
Dressel et al., 2010 [65]	1 svPPA	48	Single-case pre-post design, treatment as within-subject factor	LRT: semantic and phonological cueing hierarchies Duration: 20 sessions (4 weeks)	8	Oral naming: - Naming of trained nouns (whole words) - Naming of untrained nouns (whole words) Other measures: - fMRI data	Oral naming results: - Improvement in naming for trained items after both types of treatment - No improvement in naming for untrained items after both types of treatment Gains maintained at follow-up: Improvement in naming for trained items Other results: Changes in cortical activity, predominantly located in right superior and inferior temporal gyrus, after treatment
Henry et al., 2013 [75]	1 svPPA 1 l/phvPPA	svPPA: 60 l/phvPPA: 54	Case series pre-post design (multiple baseline design)	svPPA patient: lexical retrieval cascade treatment plus homework Duration: 8 sessions (60 min, 4 weeks) plus 20 sessions of homework (30 min, 4 weeks) followed by: Generative naming tasks plus homework Duration: 12 sessions (120 min, 2.5 weeks) plus 12 sessions of homework (60 min, 2.5 weeks) l/phvPPA patient: Modified lexical retrieval cascade treatment plus homework Duration: 6 sessions (60 min, 8 weeks) plus 18 sessions of homework (60 min, 6 weeks)	svPPA patient: 4 and 12 l/phvPPA patient: 4, 8, 12 and 24	svPPA patient: Oral naming: - Naming of trained nouns (whole words) - Naming of untrained nouns (whole words) - Naming of untrained nouns from BNT and WAB (whole words) l/phvPPA patient: Oral naming: - Naming of trained nouns (whole words) - Naming of untrained nouns (whole words) Written naming: - Written naming of trained nouns (letter accuracy) - Written naming of untrained nouns (letter accuracy)	Oral naming results in svPPA: - Improvement in naming for trained and untrained items - Improvement in naming for untrained items from BNT only after Generative naming task Gains maintained at follow-ups: - Improvement in naming for trained and untrained items Oral naming results in l/phvPPA: - Improvement in naming for trained and untrained items Gains maintained at follow-ups: - Improvement in naming for trained and untrained items Written naming results in l/phvPPA: - Improvement in naming for trained items - No improvement in naming for untrained items Gains maintained at 4-week follow-up: - Improvement in naming for trained items
Croot et al., 2015 [18]	1 nf/avPPA 1 l/phvPPA	nf/avPPA: 80 l/phvPPA: 54	Case series pre-post design	LRT: RRIPP Duration: 10 sessions (2 weeks)	nf/avPPA patient: 4 l/phvPPA patient: 36	Oral naming: - Naming of trained nouns (whole words) - Naming of untrained nouns (whole words) Generalization measure: - Word retrieval in structured interview	Oral naming results: - Improvement in naming for trained items - No improvement in naming for untrained items Generalization: - None Gains not maintained at follow-ups.
Macoir et al., 2015 [81]	1 svPPA	72	Single-case pre-post design, multiple baseline design	LRT: semantic and phonological cueing hierarchies Duration: 12 sessions (7 weeks)	4	Oral naming: - Naming of trained verbs (whole words) - Naming of untrained verbs (whole words)	Oral naming results: - Improvement in naming for trained items - No improvement in naming for untrained items Gains maintained at follow-up: Improvement in naming for trained items
Beales et al., 2016 [83]	3 svPPA 1 l/phvPPA	svPPA: 61 (7.0) l/phvPPA: 58	Case series pre-post, multiple baseline across-behaviours design	LRT: Semantic, phonological, orthographic and autobiographical cue plus homework Duration: 8 sessions (90 min, 4 weeks) plus 8 sessions of homework (30 min, 4 weeks)	4 (at 5 weeks only a svPPA patient)	Oral naming: - Naming of trained nouns, verbs and adjectives (whole words) - Naming of untrained nouns, verbs and adjectives (whole words) Generalization measures: - Discourse measures (incidence of word classes and correct information units) - Self-assessment of change measures (self-efficacy scale and word-finding questionnaire)	Oral naming results: - Improvement in naming for trained items (no differences between word classes) for three patients (2 svPPA and 1 l/phvPPA) - Improvement in naming for trained items (for nouns and adjectives, no verbs) only for a svPPA patient - Improvement in naming for untrained items (for verbs and adjectives, no nouns) only for a svPPA patient - Improvement in naming for untrained items (for only nouns) only for a svPPA patient - Improvement in naming for untrained items (for only adjectives) for two patients (1 svPPA and 1 l/phvPPA) Generalization: - Only verbs in discourse for a svPPA patient - Only adjectives in discourse for a svPPA patient - All word classes in discourse for two patients (1 svPPA and 1 l/phvPPA) - Self-efficacy scale and word-finding questionnaire for all participant Gains maintained at follow-up: - Improvement in naming for trained items for all patients - Improvement in naming for untrained items for all patients
Grasso et al., 2017 [82]	1 MCI 1 l/phvPPA	MCI: 79 l/phvPPA: 66	Single-case pre-post design (single-subject multiple baseline crossover design)	Lexical retrieval cascade treatment plus homework -MCI: Duration: 18 sessions (30 min, 9 weeks) plus 45 sessions of homework (30 min, 9 weeks) -l/phvPPA: Duration: 12 sessions (60 min, 6 weeks) plus 30 sessions of homework (30 min, 6 weeks)	12, 24 and 48	Oral naming: - Naming of trained nouns (whole words) - Naming of untrained nouns (whole words) - Naming of untrained nouns from BNT and WAB (whole words)	Oral naming results in l/phvPPA: - Improvement in naming for trained and untrained items (small effect) Gains maintained at follow-ups: - Improvement in naming for trained and untrained items
Kim, 2017 [77]	1 l/phvPPA	63	Single-case pre-post, multiple baseline crossover design	Lexical retrieval cascade treatment plus homework Duration: 8 sessions (50 min, 4 weeks) plus daily homework	20	Oral naming: - Naming of trained nouns (whole words) - Naming of untrained nouns (whole words) Generalization measure: - Discourse measures	Oral naming results: - Improvement in naming for trained items - No improvement in naming for untrained items Gains maintained at follow-up: - Improvement in naming for trained items Generalization: - Naming during scene description in one l/phvPPA patient (small effect)
Jafari et al., 2018 [66]	1 nf/avPPA	56	Single-case pre-post design, treatment as within-subjects factor	LRT: semantic and phonological cueing hierarchies vs. integrated therapy in narrative discourse context Duration: 8 sessions (60 min, 4 weeks)	2	Oral naming: - Naming of trained nouns and verbs (whole words) - Naming of untrained nouns and verbs (whole words)	Oral naming results: - Improvement in naming for both trained nouns and verbs after both types of training - Improvement in naming for both untrained nouns and verbs only after the integrated therapy Gains maintained at follow-up: -Improvement in naming for both trained nouns and verbs, although there was a decreasing trend to some extents, compared to baseline
Croot et al., 2019 [78]	3 nf/avPPA 2 svPPA 2 l/phvPPA 1 mixed PPA	nf/avPPA: 66.7 (6.8) svPPA: 64 (5.0) l/phvPPA: 61.5 (2.5) Mixed PPA: 68	Case series pre-post design	LRT: RRIPP Duration: 10-20 sessions in the first and second treatment period (2-4 weeks) and 104 sessions in the third treatment period (26 weeks)	From 9 to 84	Oral naming: - Naming of trained nouns, verbs and adjectives (whole words) - Naming of untrained nouns, verbs and adjectives (whole words)	Oral naming results: - Improvement in naming for trained items for participants that completed three periods of treatment - No improvement in naming for untrained items Gains maintained at follow-ups: - Improvement in naming for trained items
Dial et al., 2019 [84]	10 nf/avPPA (5 teletherapy; 5 FTF treatment) 10 svPPA (4 teletherapy; 6 FTF treatment) 11 l/phvPPA (5 teletherapy; 6 FTF treatment)	Script training (VISTA), Teletherapy: 67.8 (7.3) Script training (VISTA), FTF: 67.6 (3.9) LRT, Teletherapy: 61 (6.3) LRT, FTF: 68.9 (7.1)	Case series pre-post design	nf/avPPA patient: VISTA plus homework Duration: 8-12 sessions (45-60 min, 4-6 weeks) plus daily homework (30 min) svPPA and l/phvPPA patients: Lexical Retrieval Cascade Treatment (LRT1) plus homework (CART) Duration: 4-8 sessions (60 min, 4-8 weeks) plus daily homework (30 min) or modified- lexical retrieval cascade treatment (LRT2) plus homework (CART) Duration: 8-16 sessions (60 min, 4-8 weeks) plus daily homework (30 min) followed by Booster phase: 8 sessions (30 min, 4 weeks)	12, 24 and 48	nf/avPPA patients: No oral naming outcome. Primary outcome: - Writing of trained scripts (whole words) - Writing of untrained scripts (whole words) Generalization measures: - Syntactic production task (Northwestern Anagram Test) - WAB AQ svPPA and l/phvPPA: Oral naming: - Naming of trained nouns (whole words) - Naming of untrained nouns (whole words) - Naming of untrained nouns from BNT (whole words) Generalization measure: - WAB AQ	Results in nf/avPPA: - Improvement of trained scripts after both types of treatment (teletherapy or FTF) - No improvement of untrained scripts after both types of treatment (teletherapy or FTF) Generalization: None Gains maintained at follow ups: Improvement of trained scripts Oral naming results in svPPA and l/phvPPA: - Improvement in naming for trained items after both types of treatment (teletherapy or FTF) - No improvement in naming for untrained items after both types of treatment (greater effect for teletherapy) - Improvement in naming for untrained items from BNT after both types of treatment (teletherapy or FTF) Generalization: - WAB AQ after both types of treatment (teletherapy or FTF) Gains maintained at follow-ups: - Improvement in naming for trained items - Improvement in naming for untrained items until 12 weeks - Improvement in naming of untrained items from BNT until 24 weeks
Henry et al., 2019 [76]	9 svPPA 9 l/phvPPA	svPPA: 67.3 (8.7) l/phvPPA: 63.2 (7.8)	Case series pre-post design, treatment as within subjects factor	LRT1 plus homework (CART) Duration: 4-8 sessions (60 min, 4-8 weeks) plus daily homework (15 min) or LRT2 plus homework (CART) Duration: 8-16 sessions (60 min, 4-8 weeks) plus daily homework (15 min) followed by Booster phase: 8 sessions (30 min, 4 weeks)	12, 24, 48	Oral naming: - Naming of trained nouns (whole words) - Naming of untrained nouns (whole words) - Naming of untrained nouns from BNT (whole words) Generalization measures: - WAB AQ	Oral naming results: - Improvement in naming for trained items (no differences between LRT1 or LRT2 group) - Improvement in naming for untrained items (no differences between LRT1 or LRT2 group) - Improvement in naming for untrained items (BNT, grater effect after LRT1) Generalization: - None Gains maintained at 24-week follow-up: - Improvement in naming for untrained items from BNT only until 24 weeks (no differences between LRT1 or LRT2 group) Gains maintained at follow-ups: - Improvement in naming for trained items (no differences between LRT1 or LRT2 group) - Improvement in naming for untrained items (grater effects after LRT2 at 12-week follow-up)
Phonological and/or orthographic treatment							
Louis et al., 2001 [88]	3 nf/avPPA	nf/avPPA: 70.7 (5.3)	Case series pre-post design	Phonological treatment: phonological auditory tasks Duration: 42 sessions (15-20 min, 42 days)	None	Oral naming: - Naming of untrained nouns from French version of the BDAE (whole words) Generalization measures: - Written and oral comprehension tasks from BDAE - Repetition task from BDAE - Reading task from BDAE	Oral naming results: No improvement in naming for untrained items Generalization: - Written comprehension task in one patient - Repetition task in two patients - Reading task in two patients
Newhart et al., 2009 [91]	1 l/phvPPA 1 svPPA	l/phvPPA: 65 svPPA: 60	Case series pre-post design	Phonological treatment: cueing hierarchy Duration: 24-29 sessions (30-60 min, 9 weeks)	None	Oral naming: - Naming of trained nouns (whole words) - Naming of untrained nouns (whole words) Written naming: - Written naming of untrained nouns (letter accuracy) Generalization measures: - Repetition task - Spelling to dictation task - Oral reading task - Written word picture verification task	Results in l/phvPPA: Oral naming results: - Improvement in naming for trained items - Improvement in naming for untrained items (in trained categories and in untrained categories) Written naming results: - No improvement in written naming for untrained items Generalization: None Results in svPPA: Oral naming results: -Improvement in naming for trained items - No improvement in naming for untrained items (less decline for trained than untrained categories) Written naming results: - No improvement in written naming for untrained items Generalization: None
Tsapkini and Hillis, 2013 [89]	1 l/phvPPA 1 post-stroke aphasia	l/phvPPA: 62 Post-stroke aphasia: 62	Single-case pre-post design	Spelling intervention Phonemic-to-graphemic conversion treatment Duration for l/phvPPA patient: 11 sessions (60−120 min, 11 weeks) Duration for post-stroke aphasia patient: 25 sessions (60−120 min,11 weeks)	None	Written naming: - Trained phoneme-grapheme and phoneme-word correspondences (letter accuracy) - Untrained phoneme-grapheme and phoneme-word correspondences (letter accuracy) Generalization measure: - Pointing to named letters task	Written naming results in l/phvPPA: - Improvement in trained phoneme-grapheme and phoneme-word correspondences - No improvement in untrained phoneme-grapheme and phoneme-word correspondences Generalization: Pointing to named letters task
Meyer et al., 2015 [70]	1 l/phvPPA (bilingual)	69	Case series pre-post design, treatment as within-subjects factor	Phonological vs. orthographic treatment plus homework Duration: 19 sessions (48 weeks) plus 132 sessions of home practice	32, 48, 80, 144	Oral naming: - Naming of trained nouns in English and Norwegian (whole words) - Naming of untrained nouns in English and Norwegian (whole words) - Naming of untrained nouns from BNT in English and Norwegian (whole words) Written naming: - Written naming of trained nouns in English and Norwegian (letter accuracy) - Written naming of untrained nouns in English and Norwegian (letter accuracy) Generalization measure: - naming to definition (administered only at 48, 80, 144-week follow-up)	Oral naming results: - Improvement in naming for trained items after both types of treatment for English language - Improvement in naming for untrained items after both types of treatment for English language (greater effect of phonological treatment) - Improvement in naming for trained items after both types of treatment for Norwegian language (greater effect of orthographic treatment) - Improvement in naming for untrained items after both types of treatment for Norwegian language (greater effect of orthographic treatment) - No improvement in naming for untrained items from BNT Written naming results: - Improvement in written naming for trained items after both types of treatment for English language (greater effect for orthographic condition) - Improvement in written naming for untrained items after both types of treatment for English language (greater effect for orthographic condition) - Improvement in written naming for trained items after both types of treatment for Norwegian language - Improvement in written naming for untrained items after both types of treatment for Norwegian language Generalization: - Naming to definition of trained items after both types of treatment in English language - Naming to definition of untrained items after both types of treatment in English language - Naming to definition of trained items after both types of treatment in Norwegian language (greater effect for orthographic condition) - Naming to definition of untrained items after both types of treatment in Norwegian language (greater effect for orthographic condition) Gains not maintained at follow-ups.
Meyer et al., 2016 [69]	5 nf/avPPA (1 telerehabilitation; 4 FTF) 4 svPPA (1 telerehabilitation; 3 FTF) 8 l/phvPPA (1 telerehabilitation; 7 FTF)	nf/avPPA: - Tele: 48 - FTF: 67.8 (8.0) svPPA: - Tele: 68 - FTF: 63.7 (5.2) l/phvPPA: - Tele: 69 - FTF: 71.4 (7.1)	Case series pre-post design, treatment as within-subjects factor	Phonological vs. orthographic treatment: Telerehabilitation vs. FTF Duration: 68 sessions (24 weeks)	None	Oral naming: - Naming of trained nouns (whole words) - Naming of untrained nouns (whole words) Written naming: - Written Naming of trained nouns (whole words) - Written Naming of untrained nouns (whole words) Generalization measure: - Naming during scene description	Results in nf/avPPA Oral naming: - Improvement in naming for trained items (no differences between Phonological and Orthographic treatment and grater effects for Telerehabilitation participant) - No improvement in naming for untrained items Written naming results: - No improvement in written naming for trained items - No improvement in written naming for untrained items Generalization: - Naming during scene description after phonological treatment Results in svPPA Oral naming: - Improvement in naming for trained items only after phonological treatment (no differences between Telerehabilitation and FTF participants) - Improvement (marginally) in naming for untrained items only after Phonological treatment (no differences between Telerehabilitation and FTF participants) Written naming results: - No improvement in written naming for trained items - No improvement in written naming for untrained items Generalization: None Results in l/phvPPA Oral naming: - Improvement in naming for trained items (no differences between Phonological and Orthographic treatment) - Improvement (marginally) in naming for untrained items (no differences between Phonological and Orthographic treatment. Written naming results: - Improvement in written naming for trained items (greater effect after Orthographic treatment) - No improvement in written naming for untrained items Generalization: None
Meyer et al., 2017 [90]	7 nf/avPPA 5 svPPA 9 l/phvPPA	nf/avPPA: 68 (12.2) svPPA: 65.6 (5.3) l/phvPPA: 69.1 (2.3)	Case series pre-post design, treatment as a within-subject factor	Phonological vs. orthographic treatment plus homework Duration: 8 sessions (4 weeks) plus 60 sessions of home practice (20 weeks)	None	Oral naming: - Naming of trained nouns (whole words) - Naming of untrained nouns (whole words) Other measure: - Correlations between baseline brain volume and post-treatment naming accuracy	Oral naming results: -Improvement in naming for trained items after both types of treatment - Improvement in naming for untrained items after both types of treatment Other results: - Lower volume in left temporal pole is associated with decline for untrained items, while lower volume in the left inferior temporal gyrus is associated with lack of improvement for untrained items
Meyer et al., 2018a [71]	5 svPPA 9 l/phvPPA	svPPA: 65.6 (4.7) l/phvPPA: 71.1 (6.3)	Case series pre-post design, treatment as a within-subject factor	Phonological vs. orthographic treatment plus homework Duration: 13 sessions (45 min, 4 weeks) plus 60 sessions of home practice (10-15 min, 20 weeks)	None	Oral naming: - Naming of trained nouns (whole words) - Naming of untrained nouns (whole words) Written naming: - Written naming of trained nouns (whole words) - Written naming of untrained nouns (whole words) Generalization measure: - scene description task	Results in svPPA Oral naming: - Improvement in naming for trained items after both types of treatment - Improvement in naming for untrained items after both types of treatment Written naming results: -Improvement in written naming for trained items after both types of treatment (greater effect for Orthographic treatment) - No improvement in written naming for untrained items Generalization: - Scene description task after both types of treatment Results in l/phvPPA Oral naming: - Improvement in naming for trained items after both types of treatment - Improvement in naming for untrained items after both types of treatment Written naming results: - Improvement in written naming for trained items after both types of treatment (greater effect for Orthographic treatment) - No improvement in written naming for untrained items Generalization: None
Meyer et al., 2018b [72]	9 nf/avPPA 5 svPPA 12 l/phvPPA	nf/avPPA: 68.1 (9.9) svPPA: 65.6 (4.7) l/phvPPA: 69.2 (7.8)	Case series pre-post design, treatment as a within-subject factor	Phonological vs. orthographic treatment plus homework Duration: 13 sessions (45 min, 4 weeks) plus 55 sessions of home pratice (10-15 min, 20 weeks)	32 and 60	Oral naming: - Naming of trained nouns (whole words) - Naming of untrained nouns (whole words) Written naming: - Written naming of trained nouns (whole words) - Written naming of untrained nouns (whole words) Generalization measure: - scene description task	Results in nf/avPPA Oral naming: - Improvement in naming for trained items after both types of treatment (greater effect for Orthographic treatment) - Improvement in naming for untrained items after both types of treatment Written naming results: - Improvement in written naming for trained items after both types of treatment - No improvement in written naming for untrained items Generalization: - Scene description after both types of treatment (greater effect for Orthographic treatment) Gains not maintained at follow ups. Results in svPPA Oral naming: - Improvement in naming for trained items after both types of treatment (greater effect for Orthographic treatment) - Improvement in naming for untrained items after both types of treatment (greater effect for Orthographic treatment) Written naming results: - Improvement in written naming for trained items after both types of treatment - No improvement in written naming for untrained items Generalization: - Scene description task after both types of treatment (greater effect for Orthographic treatment) Gains maintained at follow ups: - Improvement in oral naming for trained items - Improvement in oral naming for untrained items - Improvement in written naming for trained items - Generalization scene description task Results in l/phvPPA Oral naming: -Improvement in naming for trained items only after orthographic treatment - Improvement in naming for untrained items only after phonological treatment Written naming results: - No improvement in written naming for trained items - No improvement in written naming for untrained items Generalization: None Gains not maintained at follow-ups.
Semantic treatment							
Snowden and Neary, 2002 [108]	2 svPPA	svPPA: 60.5 (3.5)	Case series pre-post design	Experiment 1: Relearning of names Duration: 6 sessions Experiment 2: Relearning of names with descriptive information Duration: 21 sessions (20 min, 3 weeks)	Experiment 1: 16 Experiment 2: 24	Oral naming: - Naming of trained nouns (whole words) Generalization measure (only in Experiment 2): - Picture definition task	Experiment 1 Oral naming results: - Improvement in naming for trained items - Gains not maintained at follow-up. Experiment 2 Oral naming results: - Improvement in naming for trained items Generalization: - Picture definition task Gains maintained at follow-up: - Improvement in naming for trained items - Improvement in picture definition task
Jokel et al., 2006 [105]	1 svPPA	63	Single-case pre-post design	Relearning of names Duration: 18 sessions (30 min, 3 weeks)	4 and 24	Oral naming: - Naming of trained nouns (whole words) - Naming of untrained nouns (whole words)	Oral naming results: -Improvement in naming for trained items -Little improvement in naming for untrained items Gains maintained at 4-week follow-up: - Improvement in naming for trained items Gains not maintained at 24-week follow-up.
Henry et al., 2008 [56]	2 svPPA 1 Post-stroke aphasia	svPPA: 74.5 (3.5) Post-stroke aphasia: 79	Case series pre-post multiple baseline design	Semantic treatment plus homework Duration: 12 sessions (90 min, 3 weeks) plus daily homework	16 (for 1 svPPA and post-stroke patients)	Oral naming: - Naming of untrained nouns from BNT (whole words) Generalization measure: - Generative naming task (verbal fluency) of trained categories - Generative naming task (verbal fluency) of untrained categories	Oral naming results in svPPA: - No improvement in naming for untrained items from BNT Generalization: - Generative naming task (verbal fluency) for the trained and untrained categories Gains maintained at follow-up: - Improvement on the generative naming task for the trained categories only for a svPPA patient.
Bier et al., 2009 [93]	1 svPPA	70	Single-case pre-post, treatment as within-subject factor, multiple baseline desgin	Semantic treatment with spaced retrieval vs. with simple repetition Duration: 6 sessions (3 weeks)	1, 2 and 5	Oral naming: - Naming of trained nouns (whole words) - Naming of untrained nouns (whole words) Generalization measures: - Generation of verbal attributes from spoken words for trained nouns - Generation of verbal attributes from spoken words for untrained nouns - Letter fluency task	Oral naming results: - Improvement in naming for trained items after both types of treatment - No improvement in naming for untrained items after both types of treatment Generalization: - Generation of specific verbal attributes for the trained items Gains maintained at follow-ups: - Improvement in naming for trained items
Heredia et al., 2009 [95]	1 svPPA	53	Single-case pre-post design	Relearning of names Duration: 30 sessions (4 weeks)	4 and 24	Oral naming: - Naming of trained nouns (whole words) - Naming of untrained nouns (whole words) - Naming of untrained nouns (alternative exemplars of trained nouns) (whole words)	Oral naming results: - Improvement in naming for trained items - No improvement in naming for untrained items - Improvement in naming for untrained nouns (alternative exemplars of trained items) Gains maintained at follow-ups: - Improvement in naming for trained items - Improvement in naming for untrained nouns (alternative exemplars of trained items)
Robinson et al., 2009 [107]	2 svPPA	63 (0.0)	Case series pre-post design	Errorless learning approach Duration: 4-6 sessions (2-3 weeks)	4	Oral naming: - Naming of trained nouns (whole words) - Naming of untrained nouns (whole words) Generalization measures: - Definition of trained nouns task - Definition of untrained nouns task - Object use of trained nouns task - Object use of untrained nouns task	Oral naming results: - Improvement in naming for trained items only for a subject - No improvement in naming for untrained items for both subjects Generalization: - Definition of trained items for both subjects - Definition of untrained items for a subject - Object use of trained items for a subject Gains maintained at follow-up: - Improvement in naming for trained items only for a subject - Improvement in definition of trained items for a subject - Improvement in definition of untrained items for a subject - Improvement in object use of trained items for a subject
Marcotte and Ansaldo, 2010 [99]	1 nf/avPPA 1 Post-stroke aphasia	nf/avPPA: 65 Post-stroke aphasia: 66	Single-case pre-post design	SFA therapy Duration: Nine sessions (60 min, 3 weeks)	None	Oral naming: - Naming of trained nouns and verbs (whole words) - Naming of untrained nouns and verbs (whole words) Other measure: Event-related-fMRI measures	Oral naming results in nf/avPPA: -Improvement in naming for trained items - No improvement in naming for untrained items Other results: Network expansion, mostly recruiting semantic processing areas and a gradual bilateralization of naming networks, after treatment
Jokel et al., 2010 [104]	1 svPPA	NA	Single-case pre-post design	Errorless learning approach Duration: 12 sessions (60 min, 4 weeks)	4 and 12	Oral naming: - Naming of trained nouns (whole words) - Naming of untrained nouns (whole words) - Naming of untrained nouns from BNT (whole words) - Naming of untrained nouns from PNT (whole words) Generalization measures: - Sentence production task - Semantic fluency task	Oral naming results: - Improvement in naming for trained items - Improvement in naming for untrained items - No improvement in naming for untrained items from BNT - Improvement in naming for untrained items from PNT Generalization: Semantic fluency Gains maintained at follow-ups: - Improvement in naming for trained items
Senaha et al., 2010 [103]	3 svPPA	svPPA: 62.7 (10.1)	Case series pre-post design	Relearning of names based on errorless learning approach Duration: 48-144 sessions (24-72 weeks)	None	Oral naming: - Naming of trained nouns (whole words) - Naming of untrained nouns from BNT (whole words)	Oral naming results: - Improvement in naming for trained items - No improvement in naming for untrained items from BNT
Beeson et al., 2011 [61]	1 l/phvPPA	77	Single-case pre-post, multiple baseline design	Semantic treatment plus homework Duration: 12 sessions (120 min, 2 weeks) plus daily homework (60 min)	3, 16 and 24	Oral naming: - Naming of untrained nouns from BNT and PNT (whole words) Generalization measures: - Generative naming task (verbal fluency) of trained categories - Generative naming task (verbal fluency) of untrained categories discourse production measure	Oral naming results: Improvement in naming for untrained items from BNT and PNT Generalization: - Generative naming task (verbal fluency) for the trained and untrained categories - Discourse production measures (speaking rate and number of correct information units) Gains maintained at follow-ups: - Improvement on the generative naming task for the trained categories - Improvement in naming for untrained items from BNT and PNT - Improvement on discourse production measures (speaking rate and number of correct information units)
Mayberry et al., 2011 [100]	2 svPPA	59 (6.0)	Case series pre-post design	Relearning of names at home administered by clinician over the phone Duration: 21 session (3 weeks)	4	Oral naming: - Naming of trained nouns (whole words) - Naming of untrained nouns from BNT (whole words)	Oral naming results: Improvement in naming for trained items in both subjects -Improvement in naming for untrained items only for a patient Gains maintained at follow-up: - Improvement in naming for trained items in both subjects - Improvement in naming for untrained items only for a patient
Jokel and Anderson, 2012 [97]	7 svPPA	68.3 (10.0)	Case series pre-post design, treatment as a within-subject factor	Relearning of names based on errorless vs. errorful learning and passive vs. active cues Duration: 12 sessions (60 min, 4 weeks)	4 and 12	Oral naming: - Naming of trained nouns (whole words) - Naming of untrained nouns from PNT (whole words) Generalization measures: - Oral sentence production test - Semantic fluency task - Semantic knowledge tasks (PPTT and K&D) - Written word–picture matching task - Peabody Picture Vocabulary Test	Oral naming results: - Improvement in naming for trained items (grater effects for errorless learning) - Improvement in naming for untrained items (grater effects for errorless learning) Generalization: - Semantic fluency task Gains maintained at follow-ups: - Improvement in naming for trained items - Improvement in naming for untrained items
Savage et al., 2013 [101]	4 svPPA	62.3 (5.5)	Single-subject design, using a multiple-baseline-across-behaviours	Relearning of names Duration: 30-60 min/day over 6 weeks	From 4 to 8	Oral naming: - Naming of trained nouns (whole words) - Naming of untrained nouns (whole words)	Oral naming results: - Improvement in naming for trained items (Indipendently from the duration of training, 3 or 6 weeks) - No improvement in naming for untrained items Gains maintained at follow-ups: - Improvement in naming for trained items
Savage et al., 2014 [102]	5 svPPA	61.8 (5.6)	Case series pre-post design	Relearning of names Duration: 40 sessions (30 min, 8 weeks)	None	Oral naming: - Naming of trained nouns (whole words) - Naming of untrained nouns (whole words) Generalization measures: - Video description task - Word comprehension tasks (household requests task and word picture matching task)	Oral naming results: - Improvement in naming for trained items - No improvement in naming for untrained items Generalization: - Video description task only for trained items in four patients - Word comprehension tasks (household requests task and word picture matching) only for trained items in two patients
Hoffman et al., 2015 [96]	3 svPPA	svPPA: 64,3 (0.5)	Case series pre-post design, treatment as within-subjects factor	Relearning of names Study 1: Manipulation of order of items during relearning (fixed order vs. variable order items) Duration: 15 sessions (20 min, 3 weeks) Study 2: Generalization of word learning to novel exemplars (administered over the telephone) Duration: 5 sessions (one week)	4, 16 and 28	Study 1 Oral naming: - Naming of trained nouns (whole words) - Naming of untrained nouns (whole words) Generalization measure: - Word-picture matching task Study 2 Oral naming: - Naming of trained nouns (whole words) - Naming of untrained nouns (whole words) Generalization measures: - Novel exemplars of the trained item - Semantically and visually similar exemplar of the trained item	Study 1 Oral naming results: - Improvement in naming for trained items in two patients (more successful naming for items that were trained in a variety of different orders) - No improvement in naming for untrained items Generalization: - Word-picture matching task of trained items for two patients Gains maintained at follow-ups: - Improvement in naming of trained items for two patients Study 2 Oral naming results: - Improvement in naming for trained items in two patients (no differences between ME and SE conditions) - No improvement in naming for untrained items Generalization: - Novel exemplars of the trained item Gains maintained at follow-ups: - Improvement in naming for trained items for only a subject
Suárez-González et al., 2015 [73]	1 svPPA	57	Single-case pre-post design, treatment as a within-subject factor	Naming treatment vs. Semantic treatment COEN Duration: 60 sessions (30 min,12 weeks) for each therapy	None	Oral naming: - Naming of trained nouns (whole words) - Naming of untrained nouns (whole words) Generalization measures: - Naming a visually dissimilar example task - Description-to-naming task - Naming-to-description task	Oral naming results: -Improvement in naming for trained items after both types of treatment - No improvement in naming for untrained items after both types of treatment Generalization: - Naming a visually dissimilar example task for trained items only after COEN therapy - Description-to-naming task for trained items only after COEN therapy - Naming-to-description task for trained items only after COEN therapy
Evans et al., 2016 [94]	1 svPPA	72	Single-case pre-post design	Relearning of names plus homework Duration: 24 sessions (60 min, 80 weeks) plus 240-320 sessions of homework (30 min, 80 weeks)	None	Oral naming: - Naming of trained nouns (whole words) - Naming of untrained nouns from BNT (whole words) Generalization measure: - WAB - CSB: word-picture matching - Verbal fluency (letter and semantic)	Oral naming results: -Improvement in naming for trained items - Improvement in naming for untrained items from BNT Generalization: - Verbal semantic fluency
Jokel et al., 2016 [67]	4 svPPA	61.3 (7.8)	Pre-post design, treatment as within subjects factor	Semantic treatment: Relearning of names based on errorless learning approach vs. Phonological treatment: Relearning of names based on errorless learning approach Duration: 20 sessions (60 min, 10 weeks)	None	Oral naming: - Naming of trained nouns (whole words) - Naming of untrained nouns (whole words) - Naming of untrained nouns from BNT (whole words) Generalization measures: - oral sentence production test - semantic knowledge task (PPTT- written word); - semantic and phonemic fluency - PPVT	Oral naming results: - Improvement in naming for trained items after both types of treatment (grater effects for semantic treatment) - Improvement in naming for untrained items after both types of treatment in three patients - Improvement in naming for untrained items from BNT Generalization: - Oral sentence production test in two patients - Semantic knowledge (PPTT) in three patients - Semantic and phonemic fluency in two patients - Semantic fluency only in a patient - PPVT in three patients
Krajenbrink et al., 2018 [68]	1 svPPA	60	Single-case pre-post design	Lexical retrieval therapy RRIPP vs. Semantic treatment COEN Duration: 19 sessions (10 min, 4 weeks) vs. 10 sessions (10 min, 2 weeks)	None	Oral naming: - Naming of trained nouns (whole words) - Naming of untrained nouns (whole words) Written naming: - Written naming of trained nouns (whole words) - Written naming of untrained nouns (whole words) Generalization measures: - Structured interview - Picture-word verification task	Oral naming results: - Improvement in naming for trained items only after RRIPP - No improvement in naming for untrained items after both types of treatment Written naming results: - Improvement in written naming for trained items only after RRIPP - No improvement in written naming for untrained items after both types of treatment Generalization: - Picture-word verification task only after RRIPP
Suárez-González et al., 2018 [74]	1 svPPA	62	Single-case pre-post design, treatment as within-subjects factor	Naming treatment vs. Semantic treatment COEN Duration: 7 sessions (60 min, one week)	3 and 6	Oral naming: - Naming of trained nouns (whole words) - Naming of untrained nouns (whole words) Generalisation measures: - Naming visually dissimilar pictures task - Description to naming task - Naming to description task	Oral naming results: - Improvement in naming for trained items after both types of treatment - No improvement in naming for untrained items after both types of treatment Generalization: - Naming visually dissimilar pictures after both types of treatment - Description to naming task only after COEN therapy - Naming to description task only after COEN therapy Gains maintained at 3-week follow-up: - Improvement in naming of trained items in both types of treatment Gains maintained at 6-week follow-up: - Improvement in naming of trained items only after COEN therapy
Lavoie et al., 2019 [98]	2 svPPA 3 l/phvPPA	svPPA: 70.5 (4.5) l/phvPPA: 73.3 (4.8)	Case series pre-post, multiple baseline design	Self-administered using a smart tablet Duration: 16 sessions (4 weeks)	2, 4 and 8	Oral naming: - Naming of trained nouns (whole words) - Naming of untrained nouns (whole words) Generalization measure: - Ecological conversation task	Oral naming results: - Improvement in naming for trained items in all patients - Improvement in naming for untrained items in only one l/phvPPA patient Generalization: - Ecological conversation task for three patients Gains maintained at follow-ups: - Improvement in naming for trained items in four participants (2 svPPA and 2 l/phvPPA)
Flurie et al., 2020 [109]	7 svPPA 2 l/phvPPA	svPPA: 63.4 (2.6) l/phvPPA: 67 (2.0)	Case series pre-post, multiple baseline design	SFA therapy at home administered by caregiver (3 sessions/week) and clinician (1 session/3 weeks) Duration: 288 sessions (96 weeks)	None	Oral naming: - Naming of trained nouns (whole words) - Naming of untrained nouns (whole words)	Oral naming results: - Maintenance in naming for trained items - No maintenance in naming for untrained items
Montagut et al., 2020 [106]	8 svPPA	64 (10.5)	Case series pre-post design	Errorless learning approach Duration: 16 sessions (45 min, 8 weeks)	4, 12 and 24	Oral naming: - Naming of trained nouns (whole words) - Naming of untrained nouns (whole words) Generalization measure: - Comprehension of trained nouns (whole words) - Comprehension of untrained nouns (whole words)	Oral naming results: - Improvement in naming for trained items - No improvement in naming for untrained items Gains maintained at 12-week follow-up: - Improvement in naming for trained items Generalization: None
Multimodality approach treatment							
Farrajota et al., 2012 [116]	20 PPA	Treatment group: 68 (7.8) Control group: 66.2 (7.7)	Controlled study (10 PPA in treatment group, 10 PPA in control group)	SLT plus homework Duration: 44 sessions (60 min, 44 weeks) plus homework	None	Oral naming: - Naming of untrained nouns form Snodgrass and Vanderwart test (whole words)	Oral naming results: - Improvement in naming for untrained items after treatment (less decline than control group)
Rogalski et al., 2016 [118]	31 PPA	67.2 (6.9)	Case series pre-post design	SLT plus homework Duration: 8 sessions (60 min) plus homework	24	Oral naming: - Naming of trained nouns (whole words) - Naming of untrained nouns from BNT (whole words) Generalization measures: - ASHA-FCM - CCRSA	Oral naming results: - Improvement in naming for trained items - No improvement in naming for untrained items from BNT Generalization to: - ASHA-FCM - CCCRSA Gains maintained at follow up: - Improvement in naming for trained items of ASHA-FCM - Improvement on CCRSA
Cadório et al., 2019 [115]	1 nf/avPPA 1 PPA	nf/avPPA: 60 PPA: 52	Case series pre-post, multiple baseline design	LRT and compensatory approach plus homework Duration: 244 sessions (60 min, 20 weeks) plus 60-80 sessions of homework (20 min, 3-4 weeks)	4	Oral naming: - Naming of trained nouns (whole words) - Naming of untrained nouns (whole words) - Naming of untrained nouns from Portuguese version of the Snodgrass and Vanderwart (whole words) Generalization measure: - Quality of life measure (SAQOL-39)	Oral naming results in nf/avPPA: - Improvement in naming for trained items - No improvement in naming for untrained items Gains maintained at follow-up: Improvement in naming for trained items Oral naming results in PPA: - No improvement in naming for trained items - Improvement in naming for untrained items - No Improvement in naming for untrained items from Portuguese version of the Snodgrass and Vanderwart Gains maintained at follow-up: Improvement in naming for untrained items Generalization: SAQOL-39 scale suggested that participants experienced a relatively steady quality of life after compensatory intervention.
Rebstock and Wallace, 2020 [117]	1 PPA	81	Single-case pre-post desing	SFA and multimodal comunication program Duration: 4 sessions (120 min, 7 weeks)	None	Oral naming: - Naming of trained nouns (whole words) - Naming of untrained nouns (whole words) Generalization measures: - Switching between communication modalities (communicative flexibility score and referential communucation task) - Communicative effectiveness (Listening task)	Oral naming results: - No improvement in naming for trained items - No improvement in naming for untrained items Generalization: - Switching between communication modalities (communicative flexibility score and referential communucation task) for trained items - Switching between communication modalities (communicative flexibility score and referential communucation task) for untrained items - Communicative effectiveness (Listening task)

*ASHA-FCM* American Speech–Language–Hearing Association functional communication measures, *BDAE* Boston Diagnostic Aphasia Examination, *BNT* Boston Naming Test, *CART* copy and recall treatment, *CCRSA* Communication Confidence Rating Scale for Aphasia, *COEN* COnceptual ENrichment therapy, *CSB* Cambridge Semantic Battery, *FTF* face to face, *K&D* kissing and dancing, *l/phvPPA* logopenic/phonological variant of primary progressive aphasia, *LRT* lexical retrieval treatment, *MCI* mild cognitive impairment, *min* minutes, *NA* not available, *nf/avPPA* non fluent/agrammatic variant of primary progressive aphasia, *PNT* Philadelphia Naming Test, *PPA* primary progressive aphasia, *PPTT* Pyramid and Palm Trees Test, *PPVT* Peabody Picture Vocabulary Test, *RIPP* repetition and/or reading in the presence of a picture, *SAQOL-39* Stroke and Aphasia Quality of Life Scale–39, *SD* standard deviation, *SFA* semantic feature analysis, *SLT* speech and language therapy, *svPPA* semantic variant of primary progressive aphasia, *VISTA* video-implemented script training for aphasia, *WAB* Western Aphasia Battery, *WAB AQ* Western Aphasia Battery Aphasia Quotient

## Overview of behavioural naming treatments for patients with PPA

Since the aim of this literature review was to identify behavioural naming treatments frequently used in clinical practice among patients with different variants of PPA, we divided the studies on the basis of the intervention used, including four types of treatment: (a) lexical retrieval treatment; (b) phonological and/or orthographic treatment; (c) semantic treatment; and (d) multimodality approach treatment (Table 1).

## Lexical retrieval treatment

Lexical retrieval treatment is the most common intervention performed in people with PPA to treat progressive word-finding difficulties [55]. This training utilises a hierarchy of tasks that are designed to promote a strategic recruitment of spared semantic, orthographic and phonological knowledge in order to facilitate word retrieval and encourage self-cueing, thereby improving the naming ability and increasing the content and efficiency of spontaneous speech, eventually generalizing naming skills to everyday life [77].

Some treatment techniques have been identified as lexical retrieval treatment, such as cueing hierarchies, lexical retrieval in context, and repetition and/or reading in the presence of a picture (RRIPP) [25].

The complete cueing hierarchy is based on the principles of the Lexical Retrieval Cascade Treatment. This intervention starts with semantic self-cueing techniques (superordinate, definition and semantic closure phrase) and progresses with orthographic and phonemic self-cues (identification of syllable’s number, first sound, first syllable) and repetition of the target word [77, 84]. However, in the shortened version in the study of Kim [81], questions on the autobiographical information in the semantic cue category are included without the semantic plausibility judgment step. The cueing hierarchy treatments have also been used for word retrieval in a discourse context [66, 81]. In this case, patients are requested to read a target story in order to be familiar with it and a question with minimal semantic information is asked in order to retrieve the target word. If the patient gives an incorrect answer, semantic and phonological cues and sentence completion cues will be provided [66]. In RRIPP, participants are required to name the picture presented on a computer screen, using the written and/or the spoken form as model before the picture disappear [18, 82].

In this review, 13 studies investigated the effectiveness of lexical retrieval treatment on naming abilities in a total of 73 patients with PPA (17 nf/avPPA, 28 svPPA, 27 l/phvPPA and 1 mixed PPA). Specifically, 12 studies assessed the oral naming performance using trained and untrained whole-word accuracy as measures, whereas only one study investigated both oral and written naming abilities using trained and untrained whole-word accuracy and letter accuracy, respectively. Most of the studies (*n* = 8) evaluated the effects of language training on naming nouns; only one focused on verbs, two investigated the naming accuracy for both nouns and verbs, and the other two studies evaluated the effects of language training on nouns, verbs and adjectives. Interestingly, all of the studies on lexical retrieval treatment assessed the long-term effects of the treatment with follow-up assessments (2–84 weeks).

Specifically, two studies have reported evidence of lexical retrieval treatment only in subjects with a diagnosis of nf/avPPA [66, 76], three only in patients with svPPA [65, 75, 78] and two only in patients with l/phvPPA [80, 81]. The other six studies included participants with different variants of PPA, such as nf/avPPA, svPPA, l/phvPPA and mixed PPA [18, 77, 79, 82–84] and aimed to examine the effects of lexical retrieval treatment on oral and/or written naming abilities by comparing different subtypes of PPA. Many of these studies showed little difference in benefits of the same type of intervention between the variants [18, 77, 82–84].

### nf/avPPA

In the two studies [66, 76] that only included subjects with a diagnosis of nf/avPPA, both applied the cueing hierarchy treatment, but used different cues to treat naming disorders. In particular, Jokel et al. [76] applied the computer-based phonological and orthographic cueing hierarchical therapy in two nf/avPPA patients and reported an improvement in oral naming of trained items without generalization to untrained items. However, the authors reported a generalization of the gains to the syntactic generation task and a maintenance of benefits till 4-week follow-up. In contrast, Jafari et al. [66] compared semantic and phonological cueing hierarchy treatments applied in a single word context and the integration of these methods in a narrative discourse context in one nf/avPPA patient. The results showed an improvement in oral naming abilities for trained nouns and verbs and partial maintenance after both types of training (for 2 weeks); the generalization to untrained items was evident only after the integrated therapy. This suggests that using tasks related to daily context, such as storytelling, could increase the possibility of generalization of the results to other tasks.

RRIPP is another word-retrieval treatment that has been applied by Croot et al. [18] in two individuals, one with nf/avPPA and the other with l/phvPPA. The procedure leads to an improvement in oral naming for trained items but no improvement for untrained items, and no generalization or maintenance of gains was observed at follow-up. Likewise, Croot et al. [82] further applied the same intervention in eight individuals with different PPA presentations (3 nf/avPPA, 2 l/phvPPA, 2 svPPA and 1 mixed PPA), and confirmed the effect of this treatment on trained items but with no improvement in oral naming for untrained items. In contrast to their previous study, the authors found a long-term effect of the training at follow-up.

### svPPA

Studies on the efficacy of lexical retrieval treatment to treat word-finding difficulties in participants with svPPA have reported different results. Specifically, Frattali and Kang [75] described a lexical retrieval treatment using an errorless learning approach based on Levelt’s model of lexical access [110], to treat naming difficulties in a svPPA patient, in the aim to achieve errorless in naming upon presentation of stimuli and effortful in recruitment of higher-order cognitive functions that serve to prime target words. The study showed that the oral naming of trained items was improved, but failed to show generalization effects on untrained stimuli, and gains did not persist at the 24-week follow-up. Furthermore, two studies have analysed the effects of semantic and phonological cueing hierarchy treatment on word-finding difficulties in patients with svPPA. One study compared the effects of semantic and phonological cueing hierarchy treatment on oral object-naming abilities [65], while the other study investigated the effects on verb-retrieval abilities [78]. Despite the diversity of the word classes trained, the two studies had similar results: improvement for trained items which persisted till the follow-up visit with no generalization to untrained items. Moreover, Beales et al. [79] evaluated the effects of lexical retrieval treatment using semantic, phonological, orthographic and autobiographical cues plus homework to facilitate the naming of nouns, verbs and adjectives in three patients with svPPA and one patient with l/phvPPA. The authors reported an improvement in oral naming for trained and untrained items and a generalization effect in patients with a diagnosis of svPPA. Gains were maintained during the 4-week follow-up for all participants.

### l/phvPPA

In patients with l/phvPPA, a particular technique of lexical retrieval treatment, the lexical retrieval cascade treatment, has been applied. This approach provides the use of a hierarchy of tasks that are designed to promote strategic recruitment of spared semantic, orthographic and phonological knowledge to facilitate word retrieval and to encourage self-cueing. This intervention focuses on naming ability and aims to increase the content and efficiency in spontaneous speech and generalize naming skills to different contexts of everyday life [77].

Henry et al. [77] have applied a modified version of the lexical retrieval cascade treatment combined with daily homework in a patient with l/phvPPA and a patient with svPPA. The results showed an improvement in oral naming of trained and untrained items, with maintenance of gains until 24 weeks for the patient with l/phvPPA and until 12 weeks for the patient with svPPA. Written naming, which was evaluated only in the l/phvPPA patient, showed improvement in written naming for trained but not for untrained items, with maintenance of gains at the 4-week follow-up. Henry et al. [84] have confirmed that this intervention is beneficial for word-retrieval deficits in PPA, whether caused by semantic or phonological impairment.

Dial et al. [83] further adapted the lexical retrieval cascade treatment used by Henry et al. [77] and found improved oral naming for trained and untrained items, generalization to the Western Aphasia Battery-Aphasia Quotient (WAB-AQ), and maintenance of gains at follow-up in 10 svPPA and 11 l/phvPPA patients. Interestingly, the lexical retrieval cascade treatment seems more effective when applied in the form of teletherapy than in the face-to-face approach. In the same study, the authors reported an enhancement of written naming for trained items, which was maintained at follow-up, but failed to show any generalization result in 10 nf/avPPA patients.

Grasso et al. [80] have investigated the effects of lexical retrieval cascade treatment combined with daily homework implemented by a clinician and by a trained caregiver in one patient with mild cognitive impairment (MCI) and one with l/phvPPA. They found that in the l/phvPPA patient, the treatment gains in oral naming were greater for trained items than for untrained items, and both gains were maintained at follow-up without differences between clinician-trained and caregiver-trained sets. Likewise, Kim [81] applied a lexical retrieval cascade treatment combined with daily homework to a subject with l/phvPPA. This study used a shortened cueing hierarchy, which eliminates the semantic plausibility judgement step and includes questions that prompt autobiographical information in the semantic cue category to train single-word retrieval. The treatment method was found to be effective in improving oral naming of trained words, and the maintenance of effect was evident until the 20-week follow-up. However, no improvement of generalization to untrained items was found in the l/phvPPA patient.

### Summary

In conclusion, the lexical retrieval treatment, regardless of the type, is effective in treating naming difficulties in several subtypes of PPA. Specifically, all studies have reported significant immediate gains in oral and/or written naming for trained items regardless of the grammatical class treated. However, most studies, except a few [66, 77, 79, 80, 83, 84], have failed to show larger generalization effects of a particular lexical retrieval treatment technique for untrained items when applied to a specific variant of PPA. Studies that examined this aspect have also reported effects on language tasks such as syntactic generation tasks [76] and discourse measures [79] after lexical retrieval cueing hierarchy treatment in nf/avPPA and svPPA subjects, respectively. Moreover, one study also observed a generalization effect on WAB-AQ, a measure of aphasia severity [83]. Regarding the long-term maintenance effects, generally, all strategies used for lexical retrieval treatment have led to the maintenance of gains till the end of the intervention in all subtypes of PPA. Only one study [75] did not detect a long-term effect of the treatment.

## Phonological and/or orthographic treatment

Phonologically based treatments use different methods that collectively aim to facilitate word retrieval by stimulating residual phonological representation and strengthening the phonological representation of the word [111]. Typically, in the phonological treatment a target picture is shown and a hierarchy of phonological cueing is applied using rhyming cues, first phoneme cues, and/or first syllable cues [111, 112]. Moreover, the cueing phonological hierarchy treatment can include different tasks, such as spontaneous naming, written naming, notebook search, reading and repetition. The phonological treatment has also been applied using a remediation protocol that includes auditory exercises designed to involve several aspects of phonological processing, such as phonetic and syllabic discrimination tasks and segmentation exercises [85].

In contrast, the goal of orthographic treatment is to strengthen the orthographic representation of the word. Two of the typical approaches used to achieve this goal are matching a picture with the written form of the word, thereby facilitating the participant’s access to the orthographic path to word production [70], and spelling intervention based on the phonemic-to-graphemic conversion mechanism, which requires writing of a letter or combination of letters corresponding to a particular phoneme aimed at teaching the correct phoneme-grapheme correspondence [87].

Generally, the written word-to-picture matching task is characterized by sequential presentation on a computer screen of: a target picture alone, a written word under the picture, the word alone and the written word under the picture again. Then, the participant is first asked to read and to copy the word and then to recognize images and words [69–72, 88]. In the spelling intervention, a patient is requested to write a letter or a combination of letters that correspond to a specific phoneme. Then the patient’s response is reinforced if the answer is correct, by asking him/her to write a word that starts with the trained sound; if the response is incorrect, a word beginning with that letter is provided by the therapist and the patient is asked to write that word, in order to reinforce the association between phoneme and grapheme [87].

Given the close relationship between graphemes and their corresponding phonemes, phonological and orthographic treatments have very often been compared [69–72, 88] or combined with each other [87].

Of the eight studies that investigated the effectiveness of phonological and/or orthographic treatment in a total of 85 patients with PPA (24 nf/avPPA, 20 svPPA, and 41 l/phvPPA), two studies examined the effectiveness of phonological treatment alone on naming abilities in a total of 5 patients with PPA (3 nf/avPPA, 1 svPPA, and 1 l/phvPPA), one study focused on the effects of orthographic treatment alone in a patient with l/phvPPA, while most studies (*n* = 5) compared the efficacy of orthographic and phonological treatments in a total of 79 participants with a diagnosis of PPA (21 nf/avPPA, 19 svPPA, and 39 l/phvPPA). Specifically, one study investigated oral naming performance to measure trained and untrained whole-word accuracy; one assessed oral naming performance to measure only untrained whole-word accuracy; one study assessed written naming performance as the primary outcome measure for the trained and untrained phoneme-grapheme and phoneme-word correspondences (letter accuracy); and the other five studies investigated both oral and written naming abilities using trained and untrained whole-word accuracy and letter accuracy/whole-word accuracy, respectively. All studies have evaluated the effects of language training on the naming of nouns. Interestingly, only two studies assessed the long-term effects (32–144 weeks).

Below, we will detail the evidence of gains obtained for oral and written naming abilities by application of phonological and/or orthographic treatment on different variants of PPA. Specifically, among the eight studies identified, one reported evidence only in subjects with a diagnosis of nf/avPPA [85] and two evaluated the effects of phonological and/or orthographic treatment only in patients with l/phvPPA [70, 87]. Most of the studies (*n* = 5) included participants with different PPA subtypes [69, 71, 72, 86, 88] to compare the effects of the same type of treatment on different variants of PPA.

### nf/avPPA

The study of Louis et al. [85] was the only one that investigated the application of phonological treatment on oral naming abilities only in nf/avPPA. In particular, the authors proposed a remediation protocol based on the temporal theory of phonological processes, in which phonological abilities were trained daily, and evaluated naming abilities only on untrained items. Results showed no improvements in oral naming abilities in three patients with a diagnosis of nf/avPPA. However, examination of the Boston Diagnostic Aphasia Examination profiles showed significant improvement in fluency, with a reduction in the number of phonemic paraphasias and in written comprehension (in one patient). Moreover, in two patients, there was generalization to reading and repetition tasks. The results supported the assumption that the acoustic characteristics of the auditory signal participate in the way the brain resolves phonological difficulties.

### l/phvPPA

Applying a phonological cueing hierarchy treatment in two individuals with PPA, one with l/phvPPA and one with svPPA, Newhart et al. [86] showed an improvement in both patients in oral naming abilities for trained items. In particular, there was an improvement in naming of trained and untrained items (in both trained and untrained categories) in the patient with l/phvPPA, but generalization of the gains to written naming for untrained items and to other language tasks was not found. Nevertheless, the patient with a diagnosis of svPPA did not show improvements in either oral naming or written naming for untrained items.

In contrast, the study of Tsapkini and Hillis [87] applied an orthographic treatment in a patient with l/phvPPA. The authors, using a spelling intervention, found an improvement in trained phoneme-grapheme and phoneme-word correspondence items with a generalization effect to a pointing-to-named-letters task. However, they failed to find improvement in untrained phoneme-grapheme and phoneme-word association items. Another series of studies compared the effects of orthographic and phonological treatments on naming abilities. Meyer et al. [70] compared the efficacy of orthographic and phonological treatments combined with daily homework in a bilingual patient with a diagnosis of l/phvPPA. This single-case study showed an improvement in oral naming of trained and untrained items after both types of treatment, without maintenance at follow-up (to 144 weeks). Specifically, for the English language of the bilingual patient, there was a greater effect of phonological treatment than orthographic treatment, while for the Norwegian language, the opposite pattern was found. Furthermore, both types of treatment led to an improvement in written naming for trained and untrained items. Although this study failed to find generalization effects to the Boston Naming Test (BNT), it found an improvement in naming to the definition of trained and untrained items after both types of treatment in both the English and Norwegian languages, with greater effects for orthographic conditions. In addition, Meyer et al. [69] compared the effects of phonological and orthographic treatments administered in a telerehabilitation or face-to-face manner in 5 participants with nf/avPPA, 4 with svPPA and 8 with l/phvPPA, and found improvement in oral naming for trained items in all patients. They also found a generalization to untrained items in svPPA (only after phonological treatment) and l/phvPPA patients. Moreover, an improvement in written naming abilities was recorded only in l/phvPPA patients, and only nf/avPPA patients exhibited a generalization effect to tasks that required naming abilities during scene description, but only after phonological treatment.

In a subsequent study, Meyer et al. [88] proposed the same types of treatment, administered in a face-to-face procedure, in combination with homework, in a slightly expanded study sample. Results confirmed the positive effects on oral naming abilities for trained items after both types of treatment and showed improvement in oral naming for untrained items after phonological and orthographic treatment.

In subsequent studies, the same authors [71, 72] applied the same treatment procedures to investigate the effectiveness of phonological and orthographic treatments for oral naming abilities in nf/avPPA [72], svPPA and l/phvPPA patients [71, 72]. The results showed an enhancement of oral naming of trained items in all participants, whereas generalization to oral naming of untrained items was recorded only in svPPA and l/phvPPA participants. Written naming abilities for trained items were enhanced in all participants, whereas generalization to written naming of untrained items was not evident in any of the patients.

### Summary

Phonological and orthographic treatments are frequently used in the rehabilitation of speech disorders in PPA patients. All works examined, except for the study by Louis et al. [85], described an improvement in oral naming.

Likewise, all studies that assessed written naming performance for trained items showed positive posttreatment effects of both procedures, although larger effects were generally observed using orthographic treatment [69–72, 87, 88]. Regarding generalization effects to the untrained stimuli, the results were quite variable for both oral and written naming and between variants of PPA. Specifically, most studies that assessed oral naming for untrained items have shown improvement in this outcome [69–72, 86, 88]. This effect was equally evident after phonological or orthographic treatment and generally in all subtypes of PPA, with larger effects in l/phvPPA patients. In contrast, regarding written naming, only one study reported generalization to untrained items in a l/phvPPA patient after both types of treatment [70]. Interestingly, of the eight studies on phonological and/or orthographic treatment, seven also assessed the generalization of the gains to other language tasks. In general, both types of treatment led to benefits to other language measures [69–72, 85, 87]. Regarding the long-term effects of the treatment, only two studies provided follow-up measures [70, 72], making it difficult to assess the long-term effectiveness of the phonological and orthographic treatments. Of these two studies, only one found maintenance of the gains till 60 weeks, specifically in patients with svPPA [72].

## Semantic treatment

The application of semantic treatment has been widely investigated to treat anomia in patients with PPA [57]. Semantic treatment is a privileged approach when the impairment in naming abilities is related to a deficit in the semantic system. Given the type of impairment, the aim of semantic intervention is to strengthen the association of the structural, perceptual and functional features of items, rebuilding or improving the network of information that activates them rather than strengthening semantically similar items [113]. Several types of semantic treatment have been described. Many of these are defined as semantic although the inclusion of tasks is based not only on semantic features but also on phonological or orthographical representation, aiming to strengthen the link between concepts and their corresponding names with the use of semantic feedback [68, 73, 74, 91, 92, 94, 96–103]. Generally, this treatment includes auditory word-to-picture matching, written word-to-picture matching, yes/no questions about the target answering, picture and spoken word categorization and judgment of the relatedness of target words to a series of pictures. In some studies, the picture to be named is associated with its written/auditory label or with a written/auditory description and the participant is asked to read and/or repeat the word or provide a target description, in order to reinforce its retrieval [61, 67, 89, 90, 92, 93, 95–101, 103, 105]. In addition, participants could be requested to generate semantic attributes and/or personally relevant episodic features related to the picture [56, 91, 102]. Another semantic treatment focusing on activation of semantic networks is the semantic feature analysis (SFA) training, where patients are guided by clinicians to produce semantic features of target words [94, 104]. Likewise, the COnceptual ENrichment (COEN) therapy is applied to restore concepts, which places a target picture in a personally meaningful temporal and spatial context and uses the individual’s spared semantic networks. To achieve this goal, a target image is presented together with two co-target pictures (chosen by the participant as semantically related to the target), helping to restore the meaning of the target word [68, 73, 74].

In this literature review, 23 studies have investigated the effectiveness of semantic treatment on oral and written naming abilities in a total of 66 patients with PPA (1 nf/avPPA, 59 svPPA, and 6 l/phvPPA). Specifically, 22 studies focused on oral naming performance, measuring the whole-word accuracy of items. Nineteen of these studies investigated the effects of the treatment on both trained and untrained items; one assessed the naming accuracy of only trained stimuli, while two studies examined only the naming accuracy of untrained items. Most studies (*n* = 22) examined the effects of semantic treatment training on the naming of only nouns, while one focused on the naming of nouns and verbs. In addition, more than half of the studies on semantic treatment (*n* = 15) assessed the long-term effects of the treatment with follow-ups (1–28 weeks).

Below, we detail the evidence from the literature related to the effects on oral and written naming abilities by applying semantic treatment to different variants of PPA.

Specifically, a study has reported evidence on the application of semantic treatment only in a subject with a diagnosis of nf/avPPA [94], 19 evaluated the effects of this intervention only in patients with svPPA [56, 67, 68, 73, 74, 89–93, 95–102, 105], and one focused on participants with l/phvPPA [61]. The other two studies reported patients with different variants of PPA [103, 104].

### nf/avPPA

The study by Marcotte and Ansaldo [94] was the only one that applied semantic treatment in a patient with nf/avPPA, and showed improvement in oral naming only for trained objects and verb items with nine sessions of SFA therapy. These results are interesting as they demonstrate the effectiveness of the use of semantic features to activate a semantic network, which consequently facilitates word retrieval in subjects with deficits that are primarily phonological in nature.

### svPPA

Some studies have investigated the application of semantic treatments in patients with svPPA and reported different results regarding the benefits on naming abilities of trained items, generalization to untrained items and/or other language tasks, and long-term maintenance of effects [56, 67, 68, 73, 74, 89–93, 95–105]. Regarding the studies that used traditional semantic treatments to verify the improvement of oral naming abilities on trained and untrained items, Snowden and Neary [89] applied semantic treatment based on a repeated exposure paradigm combined with home training in two patients with svPPA and reported improvement in relearning trained items. The results suggest the importance of residual semantic knowledge and the “personal meaningfulness” of selected items for the training, which may influence the learning success, generalization and maintenance of effects. In line with this, Jokel et al. [90] used a treatment programme for renaming learning in a patient with semantic dementia based on the principle that personal “meaningfulness” and personal experience of objects have implications for the ability to recognize objects and learn their names. The results suggested that the oral naming ability for objects is facilitated by intact semantic knowledge and that it contributes to a slower rate of forgetfulness, showing an improvement in oral naming of trained and untrained items for the objects that the patient was unable to name but able or unable to comprehend, associated with maintenance of improvement only for trained items at the 4-week follow-up. In addition, to monitor the progression of loss, the authors investigated the ability to generalize benefits to untrained stimuli set featuring items that the patient was able to name and comprehend before treatment. Evidence suggests that the practice delays the progression of loss. In contrast, Henry et al. [56] applied intensive semantic treatment combined with daily homework tasks aimed at improving lexical retrieval in the context of generative naming for a series of semantic categories in two cases of svPPA (and in a patient with poststroke aphasia) and reported improvement in oral naming for trained and untrained categories in both svPPA subjects and maintenance of benefits at the 16-week follow-up for trained categories in only one svPPA patient. The authors also investigated the generalization of the effects on naming abilities, but did not find improvement in this task. Bier et al. [91] compared the effects of formal semantic therapy with a simple repetition approach and formal semantic therapy combined with a spaced retrieval method on the ability to relearn names and semantic attributes of concepts in a patient with svPPA. The comparison to a simple repetition approach is important to explore the possible superiority of spaced retrieval in relearning lost names, in long-term retention and in generalization after interventions. The results showed that both types of training improved oral naming only for trained items, and the gains were maintained at follow-up, but generalization to untrained items was not found. In addition, the effects of semantic treatment were specific, since no improvement in the letter fluency task was shown, but improvement in the generation of specific verbal attributes for the trained items was evident. Similarly, Heredia et al. [92] studied the name relearning ability in a subject with svPPA using semantic relearning treatment. The results described an improvement in oral naming for trained but not for control items. Nevertheless, the patient was able to maintain the gains for 24 weeks and generalization to alternative visually similar exemplars of trained items was found. In contrast, Robinson et al. [93] examined the effectiveness of an errorless learning paradigm in improving object naming, definition and object use in two participants with svPPA. The results obtained in the two subjects were not overlaying, perhaps due to differences in the duration of the therapy and/or the severity of semantic impairment. The svPPA patient who completed all scheduled treatment sessions showed greater benefits, such as improvement in oral naming of trained items and in definition and object use for untrained and trained items, respectively, while in both subjects, improvement in definition of trained items was recorded. Differences were also detectable in long-term maintenance since only the subject who performed 3 weeks of treatment maintained the benefits. Moreover, some studies used an innovative computer-based approach to apply semantic treatment in patients with svPPA. Among these, Jokel et al. [95] investigated the effectiveness of a computer-based treatment for anomia based on the principles of errorless learning in one svPPA patient to relearn lost words (MossTalk Words) [114–116]. The authors reported improvements in oral naming for trained and untrained items after treatment, with gains for trained items maintained up to 12 weeks. Moreover, the authors found generalization of benefits to alternative visual exemplars of trained items (Philadelphia Naming Test task) and to semantic fluency task. Subsequently, Senaha et al. [96] analysed the effectiveness of semantic relearning training based on the errorless paradigm in three cases with svPPA and found improvement in oral naming focused only on trained items without generalization to untrained stimuli.

Mayberry et al. [97] applied a relearning name treatment in two svPPA patients and showed improvement in oral naming for trained items with maintenance of benefits throughout the 4-week follow-up in both subjects, but only one of the subjects showed the same results for visually dissimilar untrained items (BNT task). In contrast, Jokel and Anderson [98] investigated four treatment approaches based on the combination of errorless/errorful and passive/active learning in a sample of seven patients with semantic dementia. They reported a greater benefit for oral naming of trained and untrained items using errorless rather than errorful learning and of words for which semantic knowledge was preserved, associated with maintenance at 12-week post-treatment. The generalization effect was evident in the semantic fluency task.

Savage et al. [99] investigated a home-based method using several combinations of word relearning techniques to study possible improvement in naming abilities in four participants with svPPA. All participants showed an improvement in oral naming of trained items, showing the effectiveness of the simple treatment that pairs an object and a picture with the word form. Interestingly, the treatment was more beneficial in patients with severe semantic impairment. No improvement in oral naming was found for untrained items, but the maintenance of gains was evident for all the subjects independent of the duration of training, although a longer period of practice resulted in a reinforcement of the material learned. In a later study, the authors applied an online relearning programme in five svPPA patients with different degrees of semantic impairment, and assessed their ability to relearn items and the extension of improvements to other expressive and receptive language tasks [100]. An improvement in oral naming of trained items, but not of untrained items, was recorded in all the participants. For most of the patients, an increase in word retrieval was found for trained items on video description tasks, while generalization to word comprehension tasks was evident only in two participants with mild (not severe) disease severity. Furthermore, Hoffman et al. [101] used a name relearning intervention based on the learning theory that greater variability of experience during learning leads to more successful recall in a novel context [117, 118]. As hypothesized by the authors, manipulating the order of items during the training increased the capacity of subjects with svPPA to generalize the benefits on trained items to novel tasks (word-picture matching task). In a second study, the authors noted that varying the type of exemplars of the same object during the training sessions improved the generalization to new exemplars of the object compared with treatment based on a single exemplar of items. This evidence was found only for the oral naming ability of trained items but not for untrained items. In both studies, long-term effects were reported at 28 weeks of follow-up. Moreover, Suárez-González et al. [73] compared the effect of a standard naming treatment (NT) with COEN therapy aimed at strengthening the semantic networks of target items in a patient with svPPA. Both therapies were effective in improving the oral naming abilities of trained items, but no improvement was found for untrained items. However, they had different performance in generalization tasks, where the COEN therapy had greater effects on naming a visually dissimilar example, in the description-to-naming task and the naming-to-description task for trained items. Furthermore, in a subsequent work, the same authors [74] extended the study to another patient with svPPA to investigate the possible differences in terms of generalization and maintenance of benefits. The authors reported that the COEN therapy facilitated the generalization of tasks other than the NT therapy and permitted the maintenance of gains until 6 weeks after the training.

In a single case study, Evans et al. [102] reported an improvement in oral naming of both trained and untrained items after training. The authors suggested that the use of computer-based semantic treatment in addition to a home practice period based on trained material in a svPPA patient was effective in retaining and releasing a large set of personally meaningful words. At the end of the treatment, the patient was able to generalize the gains to BNT untrained stimuli and to semantic fluency tasks.

Interestingly, Jokel et al. [67] investigated the differences in gains in object naming abilities after application of a semantic and a phonological treatment based on principles of errorless learning in four participants with svPPA. During the semantic treatment, proven cues were derived from the meaning of trained items, while the phonological approach included phonemic cues based on the sound and form of words. The results showed that all participants improved their oral naming abilities for trained items after both types of treatment, although greater effects were reported after semantic treatment. In contrast, generalization of treatment benefits to untrained items and to other language tasks assessed as sentence production, semantic knowledge, verbal fluency and vocabulary was heterogeneous between individuals. Krajenbrink et al. [68] compared the efficacy of two home-based language therapies, a lexical retrieval therapy (RRIPP) and a semantic treatment (COEN), on oral and written naming abilities in a patient with svPPA. In the first two phases of the study, the authors compared two lexical retrieval therapies (RRIPP) based on spoken output with and without written responses, and in the third phase the semantic treatment was applied. The results suggested that regardless of the output used, the lexical retrieval therapy led to an improvement in the oral and written naming of trained items but not of untrained items. Moreover, generalization to the comprehension task was found only after the application of RRIPP with spoken and written productions. In contrast to previous studies, no improvements or generalization effects were reported after the COEN therapy.

Recently, the effectiveness of language training by errorless learning has been evaluated by Montagut et al. [105] in 8 patients with svPPA. This study showed an improvement in oral naming of trained items, and this gain was maintained at 12 weeks after intervention without any generalization result.

Finally, Flurie et al. [104] evaluated the effectiveness of a semantically based language treatment focusing on the maintenance of individualized vocabulary in patients with svPPA (*n* = 7) and l/phvPPA (*n* = 2). In this study, the use of SFA therapy induced preservation of naming ability for trained items for up to 96 weeks, in comparison to a decline for untrained items.

### l/phvPPA

The study of Beeson et al. [61] applied semantic treatment in a patient with l/phvPPA using an intensive 2-week semantically based lexical retrieval treatment, during which the participant was trained to describe the attributes of target items to strengthen the semantic links and phonological representation. Results showed that the training led to positive effects on generative naming tasks for trained and untrained categories, both after treatment and in the long term (24 weeks after the end of treatment). Moreover, a beneficial effect was shown for oral naming abilities and discourse production measures. Lavoie et al. [103] compared the effects of a self-administered semantic treatment applied to patients with svPPA and l/phvPPA. All participants showed improvement in the naming of trained items, long-term maintenance of the effects, and generalization effects, without distinction between patients, while only one l/phvPPA patient also showed an improvement in oral naming of untrained stimuli.

### Summary

The effects of semantic treatment on naming abilities have important clinical implications, especially in the treatment of anomia related to a neurodegenerative deficit in the semantic system. Although most of the studies have applied semantic treatment in subjects with svPPA and only a few investigated the effects on nf/avPPA [94] and l/phvPPA [61, 103], we generally observed that semantic intervention can lead to benefits for the treatment of word-finding difficulties in all subtypes of PPA. Again, in most studies described above, the application of this type of treatment also involves immediate posttreatment improvement in oral naming of trained items without differences between word classes or variants of PPA. Regarding the written naming, only one study investigated the effects of semantic treatment on this ability and it found no improvement for either trained or untrained items [68]. Wide variability can be found in the generalization of the effects to oral naming of untrained stimuli and to other tasks. Only eight of 20 studies that included untrained items showed a generalization effect [61, 67, 90, 95, 97, 98, 103]. In contrast, regarding generalization to other tasks, all studies that examined this effect have reported a benefit following semantic treatment to other language measures [56, 61, 67, 68, 73, 74, 89, 91, 93, 95, 98, 100–103]. Likewise, all studies that investigated the long-term effect of the treatment have shown maintenance of the gains over time [61, 74, 89, 90, 92, 93, 95, 97, 98, 101, 103–105].

## Multimodality approach treatment

Some studies investigating naming treatment in PPA have combined impairment-based approaches with functional approaches (communication skills training) to improve communicative independence in these patients. These multimodality approaches often include counselling and education on the use of strategies for patients’ caregivers [106–109].

Training involves the use of several language exercises (restorative treatments) and provides a series of strategies to help the patient express desired content (compensatory treatments). Specifically, restorative treatments are impairment-based and include tasks aimed at treating specific language deficits, such as lexical retrieval, object definition and use, and apraxia of speech. Differently, compensatory treatments involve tasks focused on developing communication strategies, such as sentence production, conversation practice, word writing, use of an Alternative and Augmentative Communication devices and role-playing, in order to increase and facilitate the individual’s participation in daily life activities [22, 119].

This type of rehabilitative approach is frequently addressed to patients not distinguished by clinical variants [106, 107, 109] since such patients are characterized by heterogeneity of language impairments.

Four studies that used multimodality approaches in a total of 54 patients with PPA were identified in our literature review. Only one of these patients received a diagnosis of nf/avPPA, while the others (*n* = 53) were not classified into a specific subtype of PPA.

All of these studies focused on the performance of oral naming using whole-word accuracy as the primary outcome. Three of the four studies examined the effects of the treatment on naming abilities for both trained and untrained nouns, while the other one assessed only the naming accuracy of untrained nouns. Finally, in only half of the studies was the maintenance of the gains evaluated through follow-up assessments (4–24 weeks).

Below, we will detail the evidence from the four studies.

In addition to the widely described benefits for a variety of language measures and quality of life in chronic aphasia [120], Farrajota et al. [106] evaluated a multimodality stimulation approach including picture naming, descriptions of pictures, auditory-verbal comprehension, reading and writing, expression of feelings and opinions, conversational skills plus homework exercises in a group of participants with PPA, in comparison to a control group of PPA patients who did not receive intervention. The results suggested a less severe decline in oral naming abilities for untrained items in patients receiving the multimodality approach, demonstrating the efficacy of the therapy in attenuating the severity of progression of language difficulties.

Moreover, Rogalski et al. [107] used a novel internet-based multimodality programme that included impairment-based approaches, participation-based approaches, ongoing disease education, counselling and support in a group of 31 PPA patients. The results showed improvement only in oral naming of trained items and a generalization effect on functional communication measures. In addition, gains were maintained throughout 24 weeks of follow-up.

Another innovative anomia treatment combines restorative and compensatory approaches to improve deficits and develop communication strategies to facilitate daily life activities of patients, as has been described by Cadório et al. [108]. This study showed an improvement in oral naming of trained items in a patient with nf/avPPA and an improvement in oral naming of untrained items in a patient with mixed PPA, with gains maintained at the 4-week follow-up and generalization of gains to quality of life.

Recently, Rebstock et al. [109] investigated the effects of a multimodality treatment that combined semantic treatment with a multimodal communication programme in a single case of PPA. The results did not show improvement in oral naming abilities but recorded a slight improvement in switching between communication modality abilities and a significant enhancement in communicative effectiveness. Despite the lack of evidence of improvement in oral naming abilities, this pilot study provides valuable information regarding the design and implementation of a multimodal intervention for subjects with PPA and suggests the importance of communication strategy training directly for caregivers in addition to combined treatment addressed to patients.

### Summary

As shown by the above studies, the application of the multimodality approach in patients with PPA does not provide clear-cut results, perhaps due to the variability of the treatments applied and/or the heterogeneity of clinical profiles of participants. In this regard, only two studies have reported an improvement in the naming of trained items after multimodality stimulation treatment [24, 107]. Likewise, only two of the four studies have shown generalization to the naming of untrained stimuli [106, 108]. Interestingly, three studies used functional communication scales or quality-of-life questionnaires as the measure of benefit generalization and reported generalization on these secondary measures [107–109]. Regarding long-term enhancement, only two studies provided follow-up assessments [107, 108], and both showed maintenance of gains.

## Discussion

This review provides a broad overview of the effectiveness of language treatments in improving oral and written naming in PPA patients. Specifically, we describe the results arising from the application of these interventions for each variant of PPA in terms of immediate benefits on naming abilities, generalization and long-term maintenance.

**Fig. 2:**
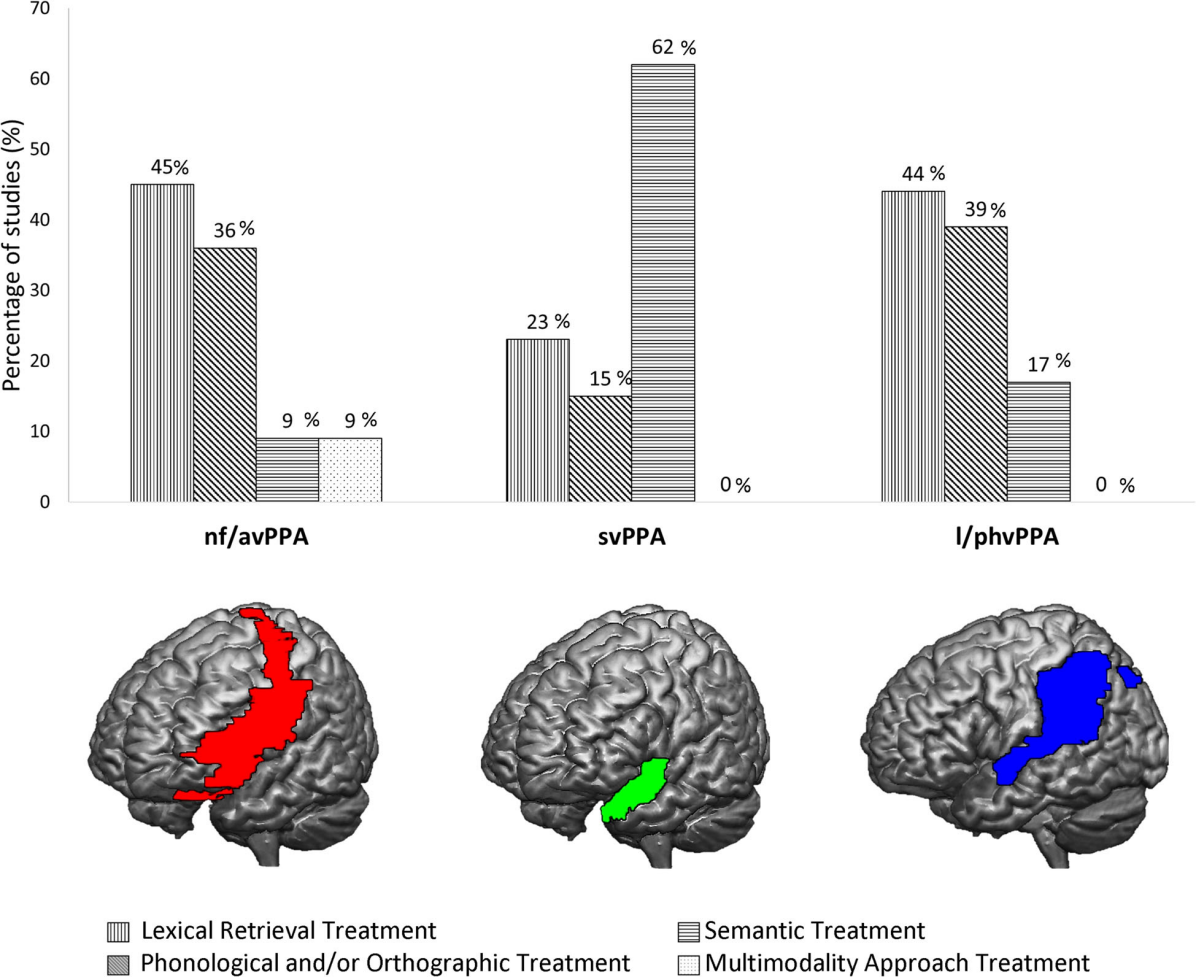
Percentage of studies that applied each type of language treatment in nonfluent/agrammatic variant (nf/avPPA, red), semantic variant (svPPA, green), and logopenic/phonological variant (l/phvPPA, blue) of PPA. Cortical areas (anatomical regions of interest were created with WFU Pickatlas (https://www.nitrc.org/projects/wfu_pickatlas/)) that can be considered as most representative for each clinical entities (nf/avPPA: left inferior frontal gyrus/insula/premotor regions; svPPA: anterior temporal lobes (mainly on the left); l/phvPPA: superior/middle temporal gyri, inferior parietal lobule) are displayed on a standardized three-dimension T1 MRI template (for template and visualization MRIcron (https://www.nitrc.org/projects/mricron) was used

Treatment of anomia can be approached with various types of integrative interventions. One well-investigated form of language intervention for PPA is lexical retrieval treatment [55]. Several treatment techniques are identified as lexical retrieval [25]; for this reason, it is used flexibly in all variants of PPA. Phonological and orthographic treatment is most frequently dedicated to patients with a diagnosis of l/phvPPA, while semantic treatment is mostly applied to svPPA patients. Finally, a number of studies have described the effects of application of a multimodal approach on the rehabilitation of anomia in PPA (Fig. 2).

Regardless of the variant of PPA, all types of language treatment seem to show significant immediate posttreatment gains in oral and/or written naming for trained items, with no significant differences among types of word class treated or among the modalities of administration of the intervention (e.g., at-home, face-to-face by clinicians or *via* telerehabilitation). Some studies included in the present review have highlighted the positive effects of telerehabilitation-based language treatment in PPA, which is a treatment modality that could also be useful to promote delivery of medical care during a large-scale epidemic, such as the coronavirus disease COVID-19 pandemic [121].

Regarding the immediate benefits for naming in PPA, interventions to rehabilitate anomia deficits are useful in patients with a speech disorder caused by a neurodegenerative disease.

Another crucial issue involves the generalization of treatment benefits to untrained stimuli, one of the more important issues in rehabilitation. Although the underlying causes of anomia are different, with the gradual loss of semantic knowledge in svPPA and the alteration of phonological processing in l/phvPPA, lexical retrieval training seems to be able to achieve generalized benefits to untrained stimuli in both variants [77, 79, 80, 83, 84]. Moreover, it seems that using a combined semantic/phonological cueing treatment in the context of narrative discourse may further increase the possibility of generalization of the results to untrained items in patients with nf/avPPA [66]. Interestingly, phonological and orthographical treatment seems to induce generalization effects to untrained stimuli in l/phvPPA patients [69–72, 86, 88], whereas semantic treatment often shows generalization effects to untrained stimuli in svPPA patients [67, 90, 92, 95, 97, 98, 102].

With respect to the possibility of eliciting generalization to other language tasks by applying language training in PPA, this result has been recorded after the application of phonological or orthographic treatment in patients with a diagnosis of svPPA and l/phvPPA [70–72, 87] and after phonological treatment in nf/avPPA patients [85]. Reacquired knowledge through semantic treatments relies on simple links between a concept and its corresponding name, which leads to item-specific gains. For this reason, the possibilities of generalization in svPPA patients seem to be limited [24]. Nonetheless, all studies that considered a generalization effect to other language measures in svPPA have reported encouraging data following semantic treatment [56, 67, 68, 73, 74, 89, 91, 93, 95, 98, 100–103].

Regarding the long-term maintenance of effects, lexical retrieval treatment [65, 66, 76–84] and semantic treatment [56, 61, 74, 89, 91–93, 95, 97–99, 101, 103, 105] generally produce maintained effects over time without differences between subtypes of PPA. In contrast, only one study evaluated the maintenance of gains after phonological and orthographic treatment in nf/avPPA, svPPA and l/phvPPA and recorded improvement maintenance at follow-up only in svPPA patients [72]. More studies are needed to confirm this finding.

Multimodality approaches have been less studied. Current evidence shows that this rehabilitation strategy has slight effects on naming, generalization and long-term measures, probably due to the variability of the treatments applied and/or the heterogeneity of the clinical profile of the samples [106–109].

Current research suggests that pathophysiologically targeted language treatment may be more successful than a “one size fits all” approach [122]. Similarly, our review suggests that factors that determine the choice of a particular approach or strategy involve the compromised components of the lexical/semantic processing system, under the consideration of possible efficacy predictors [70, 123–125], generalization and long-term maintenance of gains. An important question that remains unanswered concerns the choice of the most appropriate anomia training approach for each variant of PPA. In this regard the studies reviewed suggest that lexical retrieval treatment gives encouraging results when applied to all the three PPA variants, phonological treatment may offer gains when applied in patients with nf/avPPA, phonological/orthographic treatments are promising in patients with l/phvPPA, and semantic treatment appeared to be most beneficial for participants with svPPA.

As recently reported, native language is relevant on the phenotype and clinical presentation of PPA [126, 127]. The participants encompassed in this review include native English speakers with small exceptions (e.g. one Persian individual [66], and one Norwegian-English bilingual [70]). Bilingual aphasia literature has shown language-dependent variation in recovery [128–130]. Accordingly, it is conceivable that the effects of the treatment in patients with PPA might be influenced by the patient’s native language characteristics (e.g. transparent/opaque orthography, high/low inflected language) and by the specific difficulties of each patient (e. g. dysprosody).

Substantial evidence has supported that language treatment should begin as early as possible to maintain the naming abilities [23].

Although the results of the present review are of considerable interest, it is important to note some limitations. First, the included studies had methodological differences, such as heterogeneity of language treatments, differences in the sample size and variability in the outcome measures used.

Although the studies reviewed here focused on the enhancement of spoken and written naming abilities, the selected studies have assessed naming of nouns, verbs or adjectives, emphasizing the issue of selecting the most suitable outcome measurements on the basis of the language profile of the recruited PPA sample. Moreover, these studies recruited patients at different disease severity status. Taken together, these differences make it difficult to compare the results of the studies and to draw a clear-cut conclusion.

Moreover, most of the studies reviewed here employed case series with a pre-post design, while only one study was a controlled clinical trial. The results of randomized controlled trials will hopefully provide better information to guide future direction in rehabilitation. Furthermore, generalization of effects to untrained items and to other language tasks and the long-term maintenance of effects were not analysed in most of the included studies. These limitations imply the need for further research to investigate the effectiveness of different types of treatment in patients with PPA.

## Conclusions

Currently there are no curative treatments available for PPA. Although there are no clear guidelines for language treatment [23], a number of impairment-based approaches and compensatory strategies have been successfully used in PPA *patients*. In addition, innovative approaches, such as combination of non-invasive brain stimulation techniques with speech-language therapy, have shown promising results in facilitating naming abilities in PPA [2, 17, 21].

Our review indicates that language treatments aimed for progressive naming difficulties appear to be an excellent opportunity for intervention and should be viewed as a necessary component of care for individuals with PPA. Larger and more extensive clinical studies applying language training, alone or in combination with neuromodulation [17, 21, 131], are warranted and should be performed in future.

A multidisciplinary, person- and goal-centered approach is desirable, which highlights the importance of collaboration among professionals of different backgrounds in the field of PPA, with the overall aim of maintaining daily life functioning by improving the efficiency of treatment and patient care [13, 23]. Yet, another challenge in neurodegenerative disease management is to guarantee the continuity of care over time. In this regard telehealth-delivered speech-language interventions provide new opportunities for PPA patients who need intensive and long-lasting interventions.

## Data Availability

Data sharing is not applicable to this article as no datasets were generated or analysed during the current study.

## Abbreviations

BNT: Boston Naming Test
COEN: COnceptual ENrichment therapy
l/phvPPA: Logopenic/phonological variant of primary progressive aphasia
LRT: Lexical retrieval treatment
MCI: Mild cognitive impairment
nf/avPPA: Nonfluent/agrammatic variant of primary progressive aphasia
NT: Naming treatment
PPA: Primary progressive aphasia
RRIPP: Repetition and/or reading in the presence of a picture
SAQOL-39: Stroke and Aphasia Quality of Life Scale – 39
SD: Standard deviation
SE: Single exemplars
SFA: Semantic feature analysis
svPPA: Semantic variant of primary progressive aphasia
WAB-AQ: Western Aphasia Battery-Aphasia Quotient

## References

[1] Mesulam MM (2001). Primary progressive aphasia. Ann Neurol.

[2] Tee BL, Gorno-Tempini ML (2019). Primary progressive aphasia: a model for neurodegenerative disease. Curr Opin Neurol.

[3] Gorno-Tempini ML, Dronkers NF, Rankin KP, Ogar JM, Phengrasamy L, Rosen HJ, et al. Cognition and anatomy in three variants of primary progressive aphasia. Ann Neurol. 2004;55(3):335–46. 10.1002/ana.10825 PMC2362399 14991811

[4] Mesulam MM (1982). Slowly progressive aphasia without generalized dementia. Ann Neurol.

[5] Mesulam MM (2003). Primary progressive aphasia--a language-based dementia. N Engl J Med.

[6] Gorno-Tempini ML, Hillis AE, Weintraub S, Kertesz A, Mendez M, Cappa SF, et al. Classification of primary progressive aphasia and its variants. Neurology. 2011;76(11):1006–14. 10.1212/WNL.0b013e31821103e6 PMC3059138 21325651

[7] Olney NT, Spina S, Miller BL (2017). Frontotemporal dementia. Neurol Clin.

[8] Croot K, Ballard K, Leyton CE, Hodges JR (2012). Apraxia of speech and phonological errors in the diagnosis of nonfluent/agrammatic and logopenic variants of primary progressive aphasia. J Speech Lang Hear Res.

[9] De Leon J, Mandelli ML, Nolan A, Miller ZA, Mead C, Watson C (2019). Atypical clinical features associated with mixed pathology in a case of non-fluent variant primary progressive aphasia. Neurocase..

[10] Grossman M (2010). Primary progressive aphasia: clinicopathological correlations. Nat Rev Neurol.

[11] Mandelli ML, Caverzasi E, Binney RJ, Henry ML, Lobach I, Block N, et al. Frontal white matter tracts sustaining speech production in primary progressive aphasia. J Neurosci. 2014;34(29):9754–67. 10.1523/JNEUROSCI.3464-13.2014 PMC4099550 25031413

[12] Rahul DR, Joseph PR. Language impairment in primary progressive aphasia and other neurodegenerative diseases. J Genet. 2019;98(4). 31767822

[13] Marshall CR, Hardy CJD, Volkmer A, Russell LL, Bond RL, Fletcher PD, et al. Primary progressive aphasia: a clinical approach. J Neurol. 2018;265(6):1474–90. 10.1007/s00415-018-8762-6 PMC5990560 29392464

[14] Henry ML, Gorno-Tempini ML (2010). The logopenic variant of primary progressive aphasia. Curr Opin Neurol.

[15] Harris JM, Gall C, Thompson JC, Richardson AM, Neary D, du Plessis D (2013). Classification and pathology of primary progressive aphasia. Neurology..

[16] Montembeault M, Brambati SM, Gorno-Tempini ML, Migliaccio R (2018). Clinical, anatomical, and pathological features in the three variants of primary progressive aphasia: a review. Front Neurol.

[17] Tippett DC, Hillis AE, Tsapkini K (2015). Treatment of primary progressive aphasia. Curr Treat Options Neurol.

[18] Croot K, Taylor C, Abel S, Jones K, Krein L, Hameister I, et al. Measuring gains in connected speech following treatment for word retrieval: a study with two participants with primary progressive aphasia. Aphasiology. 2015;29(11):1265–88.

[19] Grossman M, Irwin DJ (2018). Primary progressive aphasia and stroke aphasia. Continuum (Minneap Minn).

[20] Younes K, Miller BL (2020). Neuropsychiatric aspects of frontotemporal dementia. Psychiatr Clin North Am.

[21] Cotelli M, Manenti R, Ferrari C, Gobbi E, Macis A, Cappa SF (2020). Effectiveness of language training and non-invasive brain stimulation on oral and written naming performance in primary progressive aphasia: a meta-analysis and systematic review. Neurosci Biobehav Rev.

[22] Croot K, Nickels L, Laurence F, Manning M (2009). Impairment- and activity/participation-directed interventions in progressive language impairment: clinical and theoretical issues. Aphasiology..

[23] Volkmer A, Rogalski E, Henry M, Taylor-Rubin C, Ruggero L, Khayum R, et al. Speech and language therapy approaches to managing primary progressive aphasia. Pract Neurol. 2020;20(2):154–61. 10.1136/practneurol-2018-001921 PMC6986989 31358572

[24] Cadorio I, Lousada M, Martins P, Figueiredo D (2017). Generalization and maintenance of treatment gains in primary progressive aphasia (PPA): a systematic review. Int J Lang Commun Disord..

[25] Croot K (2018). Treatment for lexical retrieval impairments in primary progressive aphasia: a research update with implications for clinical practice. Semin Speech Lang.

[26] Kirshner HS (2014). Frontotemporal dementia and primary progressive aphasia, a review. Neuropsychiatr Dis Treat.

[27] Henry ML, Hubbard HI, Grasso SM, Mandelli ML, Wilson SM, Sathishkumar MT, et al. Retraining speech production and fluency in non-fluent/agrammatic primary progressive aphasia. Brain. 2018;141(6):1799–814. 10.1093/brain/awy101 PMC5972572 29718131

[28] Kindell J, Sage K, Keady J, Wilkinson R (2013). Adapting to conversation with semantic dementia: using enactment as a compensatory strategy in everyday social interaction. Int J Lang Commun Disord..

[29] Taylor-Rubin C, Croot K, Power E, Savage SA, Hodges JR, Togher L (2017). Communication behaviors associated with successful conversation in semantic variant primary progressive aphasia. Int Psychogeriatr.

[30] Casarin FS, Branco L, Pereira N, Kochhann R, Gindri G, Fonseca RP (2014). Rehabilitation of lexical and semantic communicative impairments: an overview of available approaches. Dement Neuropsychol..

[31] Jokel R, Meltzer J, RJ D, ML D, CJ J, NE A (2017). Group intervention for individuals with primary progressive aphasia and their spouses: who comes first?. J Commun Disord.

[32] Grossman M, Ash S (2004). Primary progressive aphasia: a review. Neurocase..

[33] Budd MA, Kortte K, Cloutman L, Newhart M, Gottesman RF, Davis C, et al. The nature of naming errors in primary progressive aphasia versus acute post-stroke aphasia. Neuropsychology. 2010;24(5):581–9. 10.1037/a0020287 PMC3085899 20804246

[34] Jefferies E, Lambon Ralph MA (2006). Semantic impairment in stroke aphasia versus semantic dementia: a case-series comparison. Brain..

[35] Rohrer JD, Knight WD, Warren JE, Fox NC, Rossor MN, Warren JD (2008). Word-finding difficulty: a clinical analysis of the progressive aphasias. Brain..

[36] Indefrey P (2011). The spatial and temporal signatures of word production components: a critical update. Front Psychol.

[37] Mesulam MM, Rogalski EJ, Wieneke C, Hurley RS, Geula C, Bigio EH, et al. Primary progressive aphasia and the evolving neurology of the language network. Nat Rev Neurol. 2014;10(10):554–69. 10.1038/nrneurol.2014.159 PMC4201050 25179257

[38] Rohrer JD, Ridgway GR, Crutch SJ, Hailstone J, Goll JC, Clarkson MJ, et al. Progressive logopenic/phonological aphasia: erosion of the language network. Neuroimage. 2010;49(1):984–93. 10.1016/j.neuroimage.2009.08.002 PMC2943046 19679189

[39] Hillis AE, Oh S, Ken L (2004). Deterioration of naming nouns versus verbs in primary progressive aphasia. Ann Neurol.

[40] Hillis AE, Tuffiash E, Caramazza A (2002). Modality-specific deterioration in naming verbs in nonfluent primary progressive aphasia. J Cogn Neurosci.

[41] Gainotti G, Silveri MC, Villa G, Miceli G (1986). Anomia with and without lexical comprehension disorders. Brain Lang.

[42] Howard D, Gatehouse C (2006). Distinguishing semantic and lexical word retrieval deficits in people with aphasia. Aphasiology..

[43] Howard D, Patterson K, Franklin S, Orchard-Lisle V, Morton J (1985). Treatment of word retrieval deficits in aphasia. A comparison of two therapy methods. Brain..

[44] Lambon Ralph MA, Sage K, Roberts J (2000). Classical anomia: a neuropsychological perspective on speech production. Neuropsychologia..

[45] Ash S, McMillan C, Gunawardena D, Avants B, Morgan B, Khan A, et al. Speech errors in progressive non-fluent aphasia. Brain Lang. 2010;113(1):13–20. 10.1016/j.bandl.2009.12.001 PMC2839014 20074786

[46] Jordan LC, Hillis AE (2006). Disorders of speech and language: aphasia, apraxia and dysarthria. Curr Opin Neurol.

[47] Reilly J, Peelle JE, Antonucci SM, Grossman M (2011). Anomia as a marker of distinct semantic memory impairments in Alzheimer's disease and semantic dementia. Neuropsychology..

[48] Snowden JS, Kindell J, Thompson JC, Richardson AM, Neary D (2012). Progressive aphasia presenting with deep dyslexia and dysgraphia. Cortex..

[49] Garrard P, Rentoumi V, Gesierich B, Miller B, Gorno-Tempini ML (2014). Machine learning approaches to diagnosis and laterality effects in semantic dementia discourse. Cortex..

[50] Wilson SM, Henry ML, Besbris M, Ogar JM, Dronkers NF, Jarrold W, et al. Connected speech production in three variants of primary progressive aphasia. Brain. 2010;133(7):2069–88. 10.1093/brain/awq129 PMC2892940 20542982

[51] Rabinovici GD, Jagust WJ, Furst AJ, Ogar JM, Racine CA, Mormino EC, et al. Abeta amyloid and glucose metabolism in three variants of primary progressive aphasia. Ann Neurol. 2008;64(4):388–401. 10.1002/ana.21451 PMC2648510 18991338

[52] Migliaccio R, Boutet C, Valabregue R, Ferrieux S, Nogues M, Lehericy S (2016). The brain network of naming: a lesson from primary progressive aphasia. PLoS One.

[53] Win KT, Pluta J, Yushkevich P, Irwin DJ, McMillan CT, Rascovsky K (2017). Neural correlates of verbal episodic memory and lexical retrieval in logopenic variant primary progressive aphasia. Front Neurosci.

[54] Leyton CE, Landin-Romero R, Liang CT, Burrell JR, Kumfor F, Hodges JR, et al. Correlates of anomia in non-semantic variants of primary progressive aphasia converge over time. Cortex. 2019;120:201–11. 10.1016/j.cortex.2019.06.008 31325799

[55] Carthery-Goulart MT, da Silveira ADC, Machado TH, Mansur LL, Parente M, Senaha MLH (2013). Nonpharmacological interventions for cognitive impairments following primary progressive aphasia: a systematic review of the literature. Dement Neuropsychol.

[56] Henry ML, Beeson PM, Rapcsak SZ (2008). Treatment for lexical retrieval in progressive aphasia. Aphasiology..

[57] Jokel R, Graham NL, Rochon E, Leonard C (2014). Word retrieval therapies in primary progressive aphasia. Aphasiology..

[58] Kortte KB, Rogalski EJ (2013). Behavioural interventions for enhancing life participation in behavioural variant frontotemporal dementia and primary progressive aphasia. Int Rev Psychiatry.

[59] Rising K (2014). Treatment for lexical retrieval in primary progressive aphasia. Perspect Neurophysiol Neurogen Speech Lang Disord.

[60] Volkmer A, Spector A, Meitanis V, Warren JD, Beeke S (2020). Effects of functional communication interventions for people with primary progressive aphasia and their caregivers: a systematic review. Aging Ment Health.

[61] Beeson PM, King RM, Bonakdarpour B, Henry ML, Cho H, Rapcsak SZ (2011). Positive effects of language treatment for the logopenic variant of primary progressive aphasia. J Mol Neurosci.

[62] Rapp B, Glucroft B (2009). The benefits and protective effects of behavioural treatment for dysgraphia in a case of primary progressive aphasia. Aphasiology..

[63] Tsapkini K, Frangakis C, Gomez Y, Davis C, Hillis AE (2014). Augmentation of spelling therapy with transcranial direct current stimulation in primary progressive aphasia: preliminary results and challenges. Aphasiology..

[64] Volkmer A, Spector A, Warren JD, Beeke SJD (2020). Speech and language therapy for primary progressive aphasia: referral patterns and barriers to service provision across the UK. Dementia (London).

[65] Dressel K, Huber W, Frings L, Kümmerer D, Saur D, Mader I, et al. Model-oriented naming therapy in semantic dementia: a single-case fMRI study. Aphasiology. 2010;24(12):1537–58.

[66] Jafari S, Khatoonabadi AR, Noroozian M, Mehri A, Ashayeri H, Nickels L (2018). The effect of word retrieval therapy in primary progressive aphasia: a single-case study. Arch Neurosci.

[67] Jokel R, Kielar A, Anderson ND, Black SE, Rochon E, Graham S, et al. Behavioural and neuroimaging changes after naming therapy for semantic variant primary progressive aphasia. Neuropsychologia. 2016;89:191–216. 10.1016/j.neuropsychologia.2016.06.009 27297727

[68] Krajenbrink T, Croot K, Taylor-Rubin C, Nickels L. Treatment for spoken and written word retrieval in the semantic variant of primary progressive aphasia. Neuropsychol Rehabil. 2020;30(5):917–47. 10.1080/09602011.2018.1518780 30198389

[69] Meyer AM, Getz HR, Brennan DM, Hu TM, Friedman RB (2016). Telerehabilitation of anomia in primary progressive aphasia. Aphasiology..

[70] Meyer AM, Snider SF, Eckmann CB, Friedman RB (2015). Prophylactic treatments for anomia in the logopenic variant of primary progressive aphasia: cross-language transfer. Aphasiology..

[71] Meyer AM, Tippett DC, Friedman RB (2018). Prophylaxis and remediation of anomia in the semantic and logopenic variants of primary progressive aphasia. Neuropsychol Rehabil..

[72] Meyer AM, Tippett DC, Turner RS, Friedman RB. Long-term maintenance of anomia treatment effects in primary progressive aphasia. Neuropsychol Rehabil. 2019;29(9):1439–63. 10.1080/09602011.2018.1425146 PMC6066454 29380657

[73] Suárez-González A, Heredia CG, Savage SA, Gil-Néciga E, García-Casares N, Franco-Macías E, et al. Restoration of conceptual knowledge in a case of semantic dementia. Neurocase. 2015;21(3):309–21. 10.1080/13554794.2014.892624 24592963

[74] Suárez-González A, Savage SA, Caine D (2018). Successful short-term re-learning and generalisation of concepts in semantic dementia. Neuropsychol Rehabil..

[75] Frattali C, Kang YK (2004). An errorless learning approach to treating dysnomia. Brain Lang.

[76] Jokel R, Cupit J, Rochon E, Leonard C (2009). Relearning lost vocabulary in nonfluent progressive aphasia with MossTalk words®. Aphasiology..

[77] Henry ML, Rising K, DeMarco AT, Miller BL, Gorno-Tempini ML, Beeson PM (2013). Examining the value of lexical retrieval treatment in primary progressive aphasia: two positive cases. Brain Lang.

[78] Macoir J, Leroy M, Routhier S, Auclair-Ouellet N, Houde M, Laforce R (2015). Improving verb anomia in the semantic variant of primary progressive aphasia: the effectiveness of a semantic-phonological cueing treatment. Neurocase..

[79] Beales A, Cartwright J, Whitworth A, Panegyres PK (2016). Exploring generalisation processes following lexical retrieval intervention in primary progressive aphasia. Int J Speech Lang Pathol.

[80] Grasso SM, Shuster KM, Henry ML. Comparing the effects of clinician and caregiver-administered lexical retrieval training for progressive anomia. Neuropsychol Rehabil. 2019;29(6):866–95. 10.1080/09602011.2017.1339358 PMC5748023 28662598

[81] Kim M (2017). Effect of lexical retrieval cascade treatment on naming and discourse of individuals with logopenic variant of primary progressive aphasia (lvPPA). Clin Arch Commun Disord.

[82] Croot K, Raiser T, Taylor-Rubin C, Ruggero L, Ackl N, Wlasich E, et al. Lexical retrieval treatment in primary progressive aphasia: an investigation of treatment duration in a heterogeneous case series. Cortex. 2019;115:133–58. 10.1016/j.cortex.2019.01.009 30822613

[83] Dial HR, Hinshelwood HA, Grasso SM, Hubbard HI, Gorno-Tempini ML, Henry ML (2019). Investigating the utility of teletherapy in individuals with primary progressive aphasia. Clin Interv Aging.

[84] Henry ML, Hubbard HI, Grasso SM, Dial HR, Beeson PM, Miller BL, et al. Treatment for word retrieval in semantic and logopenic variants of primary progressive aphasia: immediate and long-term outcomes. J Speech Lang Hear Res. 2019;62(8):2723–49. 10.1044/2018_JSLHR-L-18-0144 PMC6802912 31390290

[85] Louis M, Espesser R, Rey V, Daffaure V, Di Cristo A, Habib M (2001). Intensive training of phonological skills in progressive aphasia: a model of brain plasticity in neurodegenerative disease. Brain Cogn.

[86] Newhart M, Davis C, Kannan V, Heidler-Gary J, Cloutman L, Hillis AE (2009). Therapy for naming deficits in two variants of primary progressive aphasia. Aphasiology..

[87] Tsapkini K, Hillis AE (2013). Spelling intervention in post-stroke aphasia and primary progressive aphasia. Behav Neurol.

[88] Meyer AM, Faria AV, Tippett DC, Hillis AE, Friedman RB (2017). The relationship between baseline volume in temporal areas and post-treatment naming accuracy in primary progressive aphasia. Aphasiology..

[89] Snowden JS, Neary D (2002). Relearning of verbal labels in semantic dementia. Neuropsychologia..

[90] Jokel R, Rochon E, Leonard C (2006). Treating anomia in semantic dementia: improvement, maintenance, or both?. Neuropsychol Rehabil..

[91] Bier N, Macoir J, Gagnon L, Van der Linden M, Louveaux S, Desrosiers J (2009). Known, lost, and recovered: efficacy of formal-semantic therapy and spaced retrieval method in a case of semantic dementia. Aphasiology..

[92] Heredia CG, Sage K, Ralph MAL, Berthier ML (2009). Relearning and retention of verbal labels in a case of semantic dementia. Aphasiology..

[93] Robinson S, Druks J, Hodges J, Garrard P (2009). The treatment of object naming, definition, and object use in semantic dementia: the effectiveness of errorless learning. Aphasiology..

[94] Marcotte K, Ansaldo AI (2010). The neural correlates of semantic feature analysis in chronic aphasia: discordant patterns according to the etiology. Semin Speech Lang.

[95] Jokel R, Rochon E, Anderson ND (2010). Errorless learning of computer-generated words in a patient with semantic dementia. Neuropsychol Rehabil..

[96] Senaha MLH, Brucki SMD, Nitrini R (2010). Rehabilitation in semantic dementia: study of the effectiveness of lexical reacquisition in three patients. Dement Neuropsychol..

[97] Mayberry EJ, Sage K, Ehsan S, Ralph MAL (2011). Relearning in semantic dementia reflects contributions from both medial temporal lobe episodic and degraded neocortical semantic systems: evidence in support of the complementary learning systems theory. Neuropsychologia..

[98] Jokel R, Anderson ND (2012). Quest for the best: effects of errorless and active encoding on word re-learning in semantic dementia. Neuropsychol Rehabil..

[99] Savage SA, Ballard KJ, Piguet O, Hodges JR (2013). Bringing words back to mind–improving word production in semantic dementia. Cortex..

[100] Savage SA, Piguet O, Hodges JR (2014). Giving words new life: generalization of word retraining outcomes in semantic dementia. J Alzheimers Dis.

[101] Hoffman P, Clarke N, Jones RW, Noonan KA (2015). Vocabulary relearning in semantic dementia: positive and negative consequences of increasing variability in the learning experience. Neuropsychologia..

[102] Evans WS, Quimby M, Dickey MW, Dickerson BC (2016). Relearning and retaining personally-relevant words using computer-based flashcard software in primary progressive aphasia. Front Hum Neurosci.

[103] Lavoie M, Bier N, Laforce R Jr, Macoir J. Improvement in functional vocabulary and generalization to conversation following a self-administered treatment using a smart tablet in primary progressive aphasia. Neuropsychol Rehabil. 2020;30(7):1224–54. 10.1080/09602011.2019.1570943 30714482

[104] Flurie M, Ungrady M, Reilly J (2020). Evaluating a maintenance-based treatment approach to preventing lexical dropout in progressive anomia. J Speech Lang Hear Res..

[105] Montagut N, Borrego-Écija S, Castellví M, Rico I, Reñé R, Balasa M, et al. Errorless learning therapy in semantic variant of primary progressive aphasia. J Alzheimers Dis. 2021;79(1):415–22. 10.3233/JAD-200904 33285632

[106] Farrajota L, Maruta C, Maroco J, Martins IP, Guerreiro M, de Mendonca A (2012). Speech therapy in primary progressive aphasia: a pilot study. Dement Geriatr Cogn Dis Extra.

[107] Rogalski EJ, Saxon M, McKenna H, Wieneke C, Rademaker A, Corden ME, et al. Communication bridge: a pilot feasibility study of internet-based speech-language therapy for individuals with progressive aphasia. Alzheimers Dement (N Y). 2016;2(4):213–21. 10.1016/j.trci.2016.08.005 PMC5423699 28503656

[108] Cadório I, Figueiredo D, Martins P, Cardoso R, Santos J, Lousada MJA. Combined restorative and compensatory treatment for primary progressive aphasia: a case report. Aphasiology. 2021;35(2):222–39.

[109] Rebstock AM, Wallace SE (2020). Effects of a combined semantic feature analysis and multimodal treatment for primary progressive aphasia: pilot study. Commun Disord Quarterly.

[110] Levelt WJ, Roelofs A, Meyer ASJB. Sciences b. a theory of lexical access in speech production. Behav Brain Sci. 1999;22(1):1–38 discussion 38–75. 10.1017/s0140525x99001776 11301520

[111] Madden EB, Robinson RM, Kendall DL (2017). Phonological treatment approaches for spoken word production in aphasia. Semin Speech Lang.

[112] Raymer AM, Thompson CK, Jacobs B, Le Grand HJA (1993). Phonological treatment of naming deficits in aphasia: Model-based generalization analysis. Aphasiology.

[113] Hillis AE, Prescott T (1991). Effects of separate treatments for distinct impairments within the naming process. Clinical Aphasiology.

[114] Fink R, Brecher A, Montgomery M, Schwartz M (2001). Moss talk words [computer software manual].

[115] Fink R, Brecher A, Sobel P, Schwartz M (2005). Computer-assisted treatment of word retrieval deficits in aphasia. Aphasiology..

[116] Fink RB, Brecher A, Schwartz MF, Robey RR (2002). A computer-implemented protocol for treatment of naming disorders: evaluation of clinician-guided and partially self-guided instruction. Aphasiology..

[117] Anderson JR, Bower GH (1972). Recognition and retrieval processes in free recall. Psychol Rev.

[118] Smith SM, Glenberg A, Bjork RA. Environmental context and human memory. Mem Cogn. 1978;6(4):342–53.

[119] Coelho CA, Sinotte MP, Duffy JR (2012). Schuell’s stimulation approach to rehabilitation. Language Intervention Strategies in Aphasia and Related Neurogenic Communication Disorders.

[120] Kelly H, Brady MC, Enderby P. Speech and language therapy for aphasia following stroke. Cochrane Database Syst Rev. 2010;12(5):CD000425. 10.1002/14651858.CD000425.pub2 20464716

[121] Mok VC, Pendlebury S, Wong A, Alladi S, Au L, Bath PM (2020). Tackling challenges in care of Alzheimer's disease and other dementias amid the COVID-19 pandemic, now and in the future. Alzheimers Dement.

[122] Ruksenaite J, Volkmer A, Jiang J, Johnson JC, Marshall CR, Warren JD (2021). Primary progressive aphasia: toward a pathophysiological synthesis. Curr Neurol Neurosci Rep.

[123] Cerami C, Dodich A, Greco L, Iannaccone S, Magnani G, Marcone A, et al. The role of single-subject brain metabolic patterns in the early differential diagnosis of primary progressive aphasias and in prediction of progression to dementia. J Alzheimers Dis. 2017;55(1):183–97. 10.3233/JAD-160682 PMC5115609 27662315

[124] Costa AS, Jokel R, Villarejo A, Llamas-Velasco S, Domoto-Reiley K, Wojtala J (2019). Bilingualism in primary progressive aphasia: a retrospective study on clinical and language characteristics. Alzheimer Dis Assoc Disord.

[125] Stewart JC, Cramer SC (2017). Genetic variation and neuroplasticity: role in rehabilitation after stroke. J Neurol Phys Ther.

[126] Canu E, Agosta F, Battistella G, Spinelli EG, DeLeon J, Welch AE, et al. Speech production differences in English and Italian speakers with nonfluent variant PPA. Neurology. 2020;94(10):e1062–72. 10.1212/WNL.0000000000008879 PMC7238919 31924679

[127] Weekes BSH (2020). Aphasia in Alzheimer's disease and other dementias (ADOD): evidence from Chinese. Am J Alzheimers Dis Other Dement.

[128] Khachatryan E, Vanhoof G, Beyens H, Goeleven A, Thijs V, Van Hulle MM (2016). Language processing in bilingual aphasia: a new insight into the problem. Wiley Interdiscip Rev Cogn Sci.

[129] Lorenzen B, Murray LL (2008). Bilingual aphasia: a theoretical and clinical review. Am J Speech Lang Pathol.

[130] Marrero MZ, Golden CJ, Espe-Pfeifer P (2002). Bilingualism, brain injury, and recovery: implications for understanding the bilingual and for therapy. Clin Psychol Rev.

[131] Byeon H (2020). Meta-analysis on the effects of transcranial direct current stimulation on naming of elderly with primary progressive aphasia. Int J Environ Res Public Health.

